# Synthesis, Anticancer Evaluation, and Molecular Docking of Novel Thiazolobenzimidazole–Thiazole Hybrids as Potent Colon Cancer Inhibitors

**DOI:** 10.1002/open.202500288

**Published:** 2025-07-11

**Authors:** Bader Huwaimel, Amr S. Abouzied, Magdi E. A. Zaki, Abdulwahab Alamri, Basant Farag, Saad Alqarni, Sobhi M. Gomha

**Affiliations:** ^1^ Department of Pharmaceutical Chemistry College of Pharmacy University of Ha'il Ha'il 55473 Saudi Arabia; ^2^ Medical and Diagnostic Research Center University of Ha'il Ha'il 55473 Saudi Arabia; ^3^ Department of Chemistry Faculty of Science Imam Mohammed Ibn Saud Islamic University (IMSIU) Riyadh 11623 Saudi Arabia; ^4^ Department of Pharmacology and Toxicology College of Pharmacy University of Ha'il Ha'il 55211 Saudi Arabia; ^5^ Department of Chemistry Faculty of Science Zagazig University Zagazig 44519 Egypt; ^6^ Department of Chemistry Faculty of Science Islamic University of Madinah Madinah 42351 Saudi Arabia

**Keywords:** ADMET profiling., anticancer activity, benzimidazothiazoles, HCT‐116, hydrazonoyl chlorides, molecular docking, thiazoles

## Abstract

In this study, a series of novel benzimidazothiazole–thiazole hybrids is synthesized via the condensation of 2‐(1‐(3‐methylbenzoimidazo[2,1‐b]thiazol‐2‐yl)ethylidene)hydrazine‐1‐carbothioamide with various hydrazonoyl halides and α‐halo compounds. Structural elucidation is confirmed by infrared, nuclear magnetic resonance (NMR), mass spectrometry (MS), and elemental analysis. The anticancer activities of the synthesized compounds are assessed against the HCT‐116 colon carcinoma cell line using the MTT assay, where compound **16b** exhibits the strongest cytotoxic effect (IC_50_ = 4.31 ± 1.07 μM), outperforming the reference drug doxorubicin (IC_50_ = 7.05 ± 0.49 μM). Compounds **16a**, **12**, and **10a** also demonstrate potent activity (IC_50_ < 7.1 μM). Molecular docking studies against the colon cancer protein target 6MTU reveal that these active compounds, especially **16b**, form stable interactions through key hydrogen bonding and π‐type interactions, with binding energies more favorable than doxorubicin. Additionally, in silico ADMET analysis highlights excellent absorption (up to 100%), moderate distribution, CYP450 interactions, and no predicted skin sensitization toxicity. These results position compound **16b** as a promising lead molecule for further preclinical development as a targeted colon cancer therapy.

## Graphical abstract

Novel benzimidazothiazole–thiazole hybrids are synthesized and evaluated for anticancer activity against HCT‐116 colon cancer cells. Compound **16b** shows superior cytotoxicity over doxorubicin. Molecular docking confirms strong binding to 6MTU protein, and ADMET profiling indicates favorable pharmacokinetics and low toxicity. The results highlight promising lead candidates for colon cancer drug development.

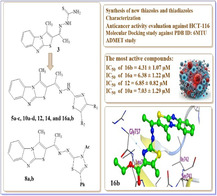

## 1 Introduction

Colon cancer, the third deadliest disease globally, accounts for ≈0.7 million deaths annually.^[^ ^1^, ^2^ ^]^ It originates from normal epithelial cells that transform into adenocarcinomas.^[^ ^3^ ^]^ Moreover, most cancer cells develop resistance mechanisms to counteract the effects of treatments.^[^ ^4^, ^5^, ^6^ ^]^ Consequently, identifying novel small molecules that target unique subcellular mechanisms is crucial for the next generation of cancer therapies.^[^ ^7^ ^]^ There remains a pressing need for new anticancer agents with greater efficacy, reduced toxicity, and minimal impact on healthy cells.^[^ ^8^ ^]^

**Figure 1:**
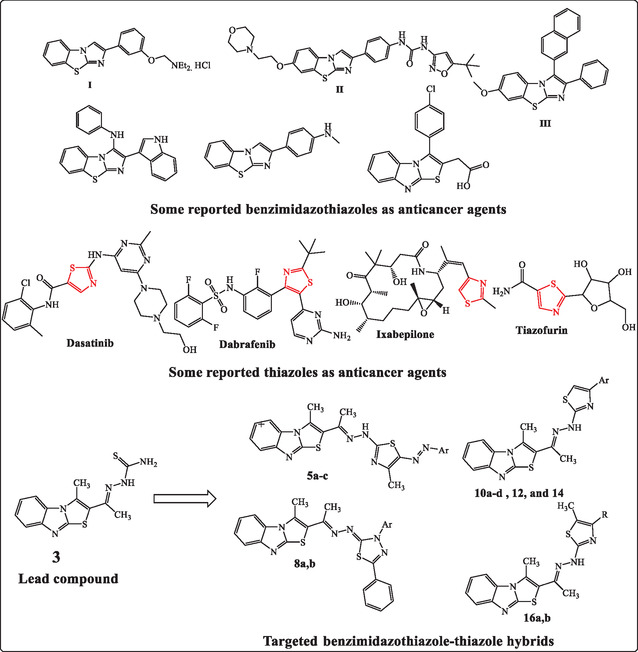
Structures of benzimidazothiazoles, thiazoles, and their derivatives as anticancer agents, highlighting targeted compounds.

Benzo[d]imidazo[2,1‐b]thiazole derivatives are fused tricyclic heterocycles containing sulfur and nitrogen, widely recognized in pharmaceutical chemistry for their bioactivity. They exhibit a broad range of biological activities, including antitumor, antimicrobial, antibacterial, antidiabetic, anti‐inflammatory, anticardiovascular, antitubercular, antineurodegenerative, immunosuppressive, and antiallergic effects.^[^ ^9^, ^10^, ^11^, ^12^, ^13^, ^14^, ^15^, ^16^, ^17^, ^18^, ^19^, ^20^, ^21^, ^22^, ^23^ ^]^ YM‐201627 (I), a fused benzo[d]imidazo[2,1‐b]thiazole compound, has been identified as a potent, orally active antitumor agent.^[^ ^24^ ^]^ Similarly, AC220 (II), a chalcone derivative, selectively inhibits FLT3 kinase and is currently in phase III clinical trials.^[^ ^25^ ^]^ Representative examples of chalcones and benzo[d]imidazo[2,1‐b]thiazole derivatives (III) are depicted in **Figure** **1**.^[^ ^26^ ^]^

On the other hand, thiazole derivatives are extensively studied in medicinal chemistry due to their diverse pharmacological properties, including antimicrobial, anti‐inflammatory, antiviral, and anticancer activities (Figure 1).^[^ ^27^, ^28^, ^29^, ^30^ ^]^ Their anticancer potential is particularly significant, as they target key biological processes involved in cancer progression, such as kinases, enzymes, and receptors.^[^ ^31^, ^32^ ^]^ Notably, thiazole‐based compounds have been identified as potent tubulin polymerization inhibitors, disrupting cell division, and as apoptosis inducers in cancer cells. Thiazolylhydrazono‐thiazoles have recently emerged as promising anticancer agents.^[^ ^33^, ^34^, ^35^ ^]^ Additionally, azolylthiazoles are key components of several FDA‐approved anticancer drugs, including ixabepilone,^[^ ^36^ ^]^ dabrafenib,^[^ ^37^ ^]^ and dasatinib.^[^ ^37^ ^]^

Building on this, recent efforts have focused on hybridizing thiazole and pyrazoline into a single framework, resulting in novel thiazolyl‐pyrazoline systems. Zhao et al. demonstrated the effectiveness of 4,5‐dihydropyrazole derivatives containing thiazole and thiophene moieties, which showed activity against WM266.4 and MCF‐7 cell lines by inhibiting BRAFV600E.^[^ ^38^ ^]^ Sadashiva and colleagues further reported the dual anticancer and antimicrobial activities of thiazole‐pyrazoline derivatives.^[^ ^39^ ^]^ Additional studies have shown the receptor tyrosine kinase inhibition of thiazoline‐pyrazolines, along with the VEGFR‐2 inhibitory activity of thiazolopyrazolyl coumarin derivatives, which exhibited minimal toxicity to normal cells.^[^ ^40^ ^]^ Vaarla et al. synthesized thiazolyl‐3‐arylpyrazole‐4‐carbaldehydes, which demonstrated cytotoxicity against HeLa cells and interacted with the cytochrome P450 enzyme.^[^ ^41^ ^]^

Furthermore, the biological activity of the synthesized compounds was analyzed, followed by an in silico docking study. Computational techniques, particularly molecular docking, play a growing role in drug development.^[^ ^42^ ^]^ This approach models interactions between compounds and target proteins, supporting in vitro assay results. In this study, the protein with PDB ID (6MTU) was targeted, and the synthesized compounds were docked to assess their potential as anti‐colon cancer agents. The study also incorporated ADMET analysis, a critical aspect of drug development. Absorption refers to how a drug enters systemic circulation, while distribution describes its movement to tissues outside the bloodstream. Metabolism involves enzymatic processing of the drug into metabolites for elimination, and excretion is the removal of these substances via urine or bile.^[^ ^43^ ^]^ Toxicology, focusing on harmful effects on humans, is increasingly emphasized in early‐stage drug research due to the growing importance of ADMET properties.^[^ ^44^, ^45^, ^46^ ^]^

Building on the promising anticancer properties of benzimidazothiazole and thiazole derivatives, their significance in drug development, and our ongoing research on bioactive heterocycles,^[^ ^38^, ^39^, ^40^, ^41^, ^47^, ^48^, ^49^, ^50^, ^51^, ^52^, ^53^ ^]^ this study aims to design, synthesize, and evaluate novel benzimidazothiazole‐thiazole hybrids for their anticancer activity against the HCT‐116 colon carcinoma cell line. The hybrids were analyzed through molecular docking studies and tested in vitro for cytotoxic effects against HCT‐116 colon cancer cells (Figure 1).

## 2 Results and Discussion

### 2.1 Chemistry

**Scheme 1:**
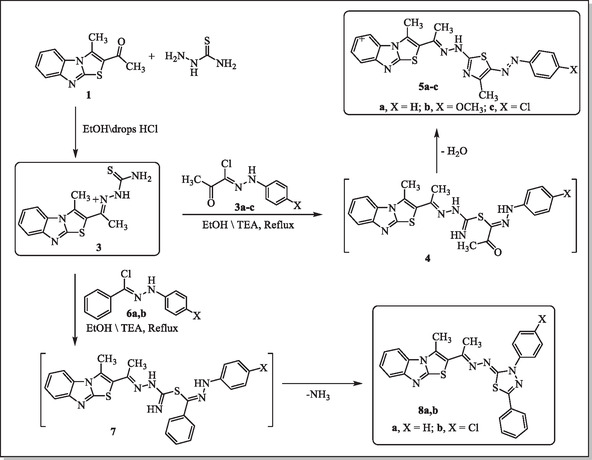
Synthesis of thiazoles **5a‐c** and thiadiazoles **8a,b**.

In our study, we synthesized 2‐(1‐(3‐methylbenzo[4‐5]imidazo[2,1‐b]thiazol‐2‐yl) ethylidene)hydrazine‐1‐carbothioamide (**3**) as a key intermediate. This compound was obtained by reacting 1‐(3‐methylbenzo[4‐5]imidazo[2,1‐b]thiazol‐2‐yl)ethan‐1‐one (**1**) with thiosemicarbazide (**2**), following the previously reported method,^[^ ^54^ ^]^ as illustrated in **Scheme** **1**.

Compound **3** was used as a versatile precursor for constructing 1,3‐thiazole and 1,3,4‐thiadiazoles. Subsequently, the reaction of thiosemicarbazone derivative **3** with 2‐oxo‐N‐arylpropanehydrazonoyl chlorides **3a‐c** yielded intermediate **4**. This transformation likely occurs through substitution at the thio group of the thiosemicarbazone scaffold. Intermediate **4** underwent cyclization, producing thiazole derivatives **5a‐c** *via* intramolecular nucleophilic attack and the elimination of water, with moderate to high yields (75%–82%) (Scheme 1).

The synthesized compounds **5a‐c** were characterized using IR, nuclear magnetic resonance (NMR), and mass spectrometry, confirming their structural integrity. IR spectra showed characteristic bands for NH (3318–3361 cm^−1^), C‐H (2927–3059 cm^−1^), and C=N (1600–1607 cm^−1^) groups. The ^1^H NMR spectra revealed signals for methyl protons (*δ* = 2.17–2.57 ppm), aromatic protons (*δ* = 6.95–7.98 ppm), and NH as a singlet (*δ* = 10.61–11.73 ppm), with unique signals such as OCH_3_ in **5b** (*δ* = 3.79 ppm). The ^1^ ^3^C NMR confirmed the presence of methyl (*δ* = 9.0–14.8 ppm) and aromatic carbons (*δ* = 104.8–159.7 ppm). Mass spectrometry provided molecular ion peaks at *m/z* = 445 (**5a**), 475 (**5b**), and 480/482 (**5c**), consistent with their calculated molecular weights, with **5c** displaying the expected isotopic pattern for chlorine.

The reaction of thiosemicarbazone derivative **3** with N‐phenylbenzohydrazonoyl chloride **6a,b** successfully yielded compounds **8a** and **8b** through the elimination of ammonia, resulting in stabilized heterocyclic structures (Scheme 1). Compounds **8a** and **8b** were obtained as yellow solids with yields of 77% and 79%, respectively. The ^1^H NMR spectra showed methyl groups at *δ* 2.38–2.47 ppm and aromatic protons at *δ* 7.07–7.97 ppm, with integration consistent with the proposed structures. Mass spectrometry confirmed the molecular formulas, displaying molecular ion peaks at m/z 480 for **8a** and m/z 515/517 for **8b**, with the latter showing the characteristic chlorine isotopic pattern.

**Scheme 2:**
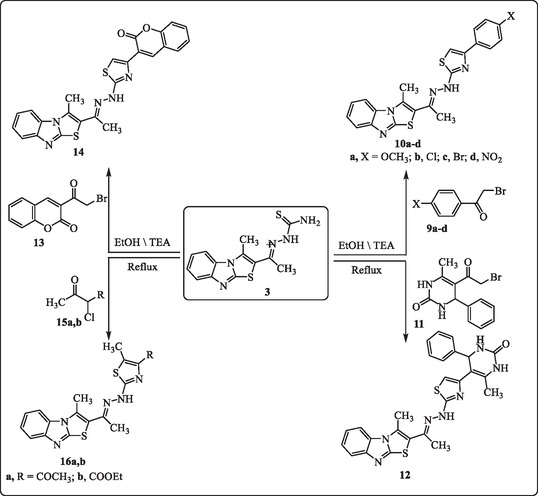
Synthesis of thiazoles **10a‐d, 12, 14,** and **16a,b**.

The synthetic strategy previously utilized for cyclization reactions was successfully adapted to α‐haloketones. Carbothioamide derivative **3** was reacted with 2‐bromo‐1‐arylethan‐1‐ones (**9a‐d**, **11**, and **13**) in ethanol under reflux conditions using triethylamine as a base catalyst. This reaction yielded hydrazonothiazoles (**10a‐d**, **12,** and **14**), as outlined in **Scheme** **2**. The resulting products were characterized through elemental analysis and various spectroscopic techniques.

The structure of compound **10a** was analyzed as a representative example. Its composition and functional groups were confirmed through spectroscopic data. The IR spectrum displayed characteristic bands at 3335 cm^−1^ (NH), 3061 and 2927 cm^−1^ (aromatic and aliphatic C‐H), and 1611 cm^−1^ (C=N). The ^1^H NMR spectrum showed singlets at *δ* 2.27 and 2.38 ppm for two methyl groups (CH_3_), *δ* 3.79 ppm for a methoxy group (OCH_3_), and a multiplet between *δ* 7.17–7.95 ppm for aromatic and thiazole protons (9H), with a deshielded singlet at *δ* 10.62 ppm corresponding to the NH proton. The ^13^C NMR spectrum supported the structural assignment, showing methyl carbon signals at *δ* 10.6 and 14.8 ppm, along with aromatic and C=N carbons between *δ* 107.6 and 163.3 ppm. Mass spectrometry confirmed the molecular formula with a molecular ion peak at m/z 433 (M^+^, 29%) and a base peak at m/z 127 (100%), aligning with the compound's molecular weight and expected fragmentation pattern.

Finally, thiosemicarbazone compound **3** was reacted with 3‐chloropentane‐2,4‐dione (**15a**) and ethyl 2‐chloroacetate (**15b**) under reflux in ethanol in the presence of catalytic amounts of triethylamine (TEA), yielding the thiazole derivatives **16a** and **16b**. The structures of the newly synthesized compounds were elucidated using spectroscopic and analytical techniques, including IR, NMR, and mass spectrometry. The IR spectra revealed characteristic NH bands at 3353 cm^−1^ for **16a** and 3347 cm^−1^ for **16b**, C‐H stretching vibrations at 3047–2925 cm^−1^, C=O bands at 1704 cm^−1^ (**16a**) and 1723 cm^−1^ (**16b**), and C=N bands at 1607–1609 cm^−1^. In the ^1^H NMR spectra, compound **16a** exhibited four methyl singlets between *δ* 2.27 and 2.57 ppm, whereas compound **16b** showed a triplet, two singlets, and a quartet in the range of *δ* 1.37–4.27 ppm. Both compounds presented aromatic multiplets (*δ* 7.25–7.71 ppm) and NH singlets (*δ* 10.69 ppm for **16a** and *δ* 10.66 ppm for **16b**). Mass spectrometry analysis confirmed the molecular ion peaks at *m/z* 383 (**16a**) and *m/z* 413 (**16b**). Stable fragment peaks at *m/z* 118 (**16a**) and *m/z* 139 (**16b**) further corroborated the proposed structures.

### 2.2 Cytotoxic Activity

Synthesized compounds were evaluated for inhibitory effects on the HCT‐116 colon carcinoma cell line, using doxorubicin as a reference. Dose‐response curves were generated for each compound, and IC_50_ values, representing the concentration needed to inhibit 50% of cell growth, were calculated. These values were averaged from three independent experiments for accuracy.

As shown in **Table** **1**, the compounds exhibited a range of cytotoxic activity, with some demonstrating strong inhibitory effects comparable to or even exceeding those of doxorubicin. The variation in IC_50_ values suggests that structural differences among the compounds play a significant role in their potency. These findings highlight the potential of the synthesized compounds as promising candidates for anticancer drug development, warranting further investigation.

**Table 1:** The impact of the tested compounds on HCT‐116 cell lines was measured using IC_50_ values, with results presented as the mean ± standard error.

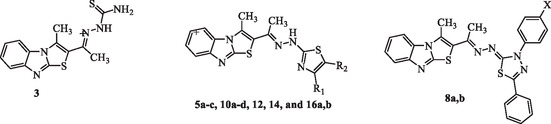				
Compound	R_1_	R_2_	X	IC_50_ values* [μM]
**3**	–	–	–	30.62 ± 2.17
**5a**	CH_3_	C_6_H_5_N=N‐	–	9.05 ± 2.01
**5b**	CH_3_	4‐CH_3_OC_6_H_4_N=N	–	8.27 ± 1.72
**5c**	CH_3_	4‐ClC_6_H_4_N=N‐	–	17.02 ± 2.00
**8a**	–	–	H	51.28 ± 2.16
**8b**	–	–	Cl	73.21 ± 1.37
**10a**	4‐CH_3_OC_6_H_4_	H	–	7.03 ± 1.29
**10b**	4‐ClC_6_H_4_	H	–	17.32 ± 1.41
**10c**	4‐BrC_6_H_4_	H	–	14.61 ± 2.23
**10d**	4‐NO_2_C_6_H_4_	H	–	27.23 ± 2.37
**12**	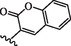	H	–	6.85 ± 0.82
**14**	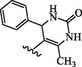	H	–	9.21 ± 1.04
**16a**	CH_3_	COCH_3_	–	6.38 ± 1.22
**16b**	CH_3_	COOEt	–	4.31 ± 1.07
Dox	–	–	–	7.05 ± 0.49

*IC_50_ values are expressed as mean ± standard error from three independent experimentsI C50 (µM): 1–8 (interesting results); 9–15 (good); 16–30 (moderate); 31–40 (poor) and above 40 (inactive).

### 2.3 Structure‐Activity Relationship (SAR)

The cytotoxic activity of the synthesized compounds against the HCT‐116 colon carcinoma cell line demonstrated notable patterns associated with variations at the R1 and R2 positions. The IC_50_ values provided in Table 1 illustrate the impact of these structural modifications on the anticancer potential of the compounds. The SAR analysis can be summarized as follows.

Among the compounds, **16b** (4.31 ± 1.07 μM) emerged as the most potent, surpassing the benchmark doxorubicin (7.05 ± 0.49 μM), followed closely by **16a** (6.38 ± 1.22 μM), **12** (6.85 ± 0.82 μM), and **10a** (7.03 ± 1.29 μM). These compounds fall into the “interesting” range (1–8 μM), indicating significant potential for further development.

Compounds **5a** (9.05 ± 2.01 μM), **5b** (8.27 ± 1.72 μM), and **14** (9.21 ± 1.04 μM) demonstrated good activity (9–15 μM), with **5b** nearing the threshold of highly potent compounds. Moderate activity (16–30 μM) was observed in compounds such as **5c** (17.02 ± 2.00 μM), **10b** (17.32 ± 1.41 μM), and **10c** (14.61 ± 2.23 μM), while compounds **3** (30.62 ± 2.17 μM), **8a** (51.28 ± 2.16 μM), and **8b** (73.21 ± 1.37 μM) were classified as poor or inactive (>30 μM). Structural modifications influenced the potency significantly, with electron‐donating groups, such as CH_3_O in **10a**, enhancing activity, while electron‐withdrawing groups like Cl and Br in **10b** and **10c**, respectively, led to a decrease. Overall, compounds **16b**, **16a**, **12**, and **10a** stand out as promising candidates for colon cancer treatment due to their superior potency and strong anticancer potential.

The superior activity of compound **16b** (IC_50_ = 4.31 ± 1.07 μM) can be attributed to the presence of an ethyl ester moiety, which may enhance cell permeability and binding affinity. Similarly, compound **16a** with a methyl ketone group also showed strong potency (IC_50_ = 6.38 ± 1.22 μM). These findings are supported by docking analysis, where both compounds formed stable hydrogen bonds within the active site of the 6MTU protein, particularly involving nitrogen atoms in the thiazole ring and residues like Arg737 and Gly739. The lower activity of compounds bearing bulky or strongly electron‐withdrawing groups (e.g., **10d**, **8b**) suggests steric hindrance or reduced interaction potential. This consistent correlation across in vitro, SAR, and docking data underscores the impact of electronic and steric features on biological efficacy.

### 2.4 Molecular Docking Study

To further investigate the binding interactions of the most active compounds against the HCT‐116 colon carcinoma cell line, a molecular docking study was performed using the target protein (PDB ID: 6MTU) and the selected ligands (**3, 5a‐c, 10a‐d, 12, 14,** and **16a, b**). Molecular docking, a computational technique, was utilized to explore the binding modes of these ligands within the active site of the protein.^[^ ^55^, ^56^ ^]^

**Figure 2:**
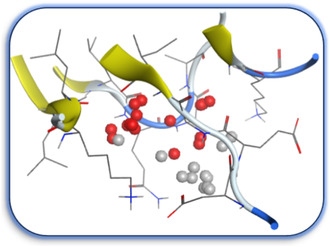
Prepared colon cancer protein (largest pocket).

The docking results were compared to doxorubicin, used as a reference ligand, and revealed the following observations: i) The binding energies of the ligands were more negative or comparable to doxorubicin, indicating enhanced or similar stability within the binding pocket. ii) The interactions involved various types, such as hydrogen bond acceptors, π‐cation interactions, π‐hydrogen bonds, and hydrogen bond donors. iii) Key amino acid residues involved in binding included glycine, arginine, serine, isoleucine, and proline. iv) The ligation primarily targeted specific regions, including sulfur (S), the imidazo[2,1‐b]thiazole scaffold, phenyl rings, and nitrogen (N) and oxygen (O) atoms. (v) Among the tested compounds, **16b, 16a, 12,** and **10a** exhibited the strongest potential for inhibition of the 6MTU protein. These results align with the in vitro findings and provide a deeper understanding of the binding mechanisms, highlighting the interactions responsible for the compounds’ inhibitory activity (**Figure** **2**). The docking results revealed distinct hydrogen bonding interactions for the tested compounds with the active site residues, as summarized in **Table** **2**. Compound **3** formed a single hydrogen bond acceptor interaction between its sulfur atom and Gly737. Compound **5b** exhibited three hydrogen bonds, including two H‐bond donors involving the sulfur atom of the thiazole ring with Ile740 and one H‐bond acceptor between the nitrogen atom and Gly739. The 3D model of compound 5c displayed one hydrogen bond acceptor interaction between the nitrogen of the imidazole ring and Gly737. For compound **10a**, one H‐bond acceptor interaction was observed between the nitrogen atom of the thiazole ring and Gly739. Similarly, compounds **10b** and **10c** each formed one hydrogen bond acceptor interaction with Arg733. Compound **14** interacted through a single hydrogen bond acceptor with Ile742. Compound **16a** demonstrated one hydrogen bond with Gly739, while compound **16b** exhibited a hydrogen bond acceptor interaction between the nitrogen atom of the thiazole ring and Arg737. As shown in **Table** **3**, these findings highlight the hydrogen bonding patterns and diverse binding modes of the tested compounds at the active site.

**Table 2:** Docking scores, H‐bonds, and arene interactions of synthesized compounds (3, 5a‐c, 10a‐d, 12, 14, 16a,b) with 6MTU versus Dox.

Compound	Docking score [kcal/mol]	Hydrogen bonding	Donor atom	Acceptor atom	NO. of arene interaction
**3**	−4.97	1 (Gly737)	–	S	1 (Pi‐Cation) [Arg762]
**5a**	−5.82	–	–	–	2 (π‐H) [Ser741] 1 (π‐H) [Gly767] 1 (π‐H) [Arg771]
**5b**	−5.83	2 (Ile740)	S	–	1 (π‐H) [Ser764]
		1 (Gly739)	–	N	
**5c**	−5.67	1 (Gly737)	–	N	2 (π‐H) [Arg733]
**10a**	−5.91	1 (Gly739)	–	N	1 (π‐H) [Pro768]
**10b**	−5.64	1 (Arg733)	–	N	2 (π‐H) [Arg801]
**10c**	−5.74	1 (Arg733)	–	N	2 (π‐H) [Arg801] 1 (π‐H) [Ser741]
**10d**	−5.64	–	–	–	1 (π‐H) [Gly739]
**12**	−6.19	–	–	–	2 (π‐H) [Ser741] 1 (π‐H) [Ile742]
**14**	−5.81	1 (Ile742)	–	O	2 (π‐H) [Ser764]
**16a**	−6.20	1 (Gly739)	–	N	1 (π‐H) [Ile740]
**16b**	−6.72	1 (Gly737)	–	N	–
**Doxorubicin**	−6.01	1 (Arg733)	–	O	–

**Table 3:** 3D and 2D ligand interactions within the 6MTU binding site for the most potent compounds (3, 5a‐c, 10a‐d, 12, 14, and 16a,b).

Cpd	3D Ligand interactions	2D Ligand interactions
**3**	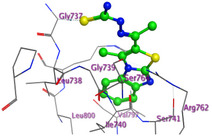	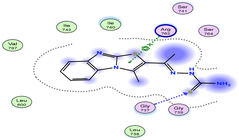
**5a**	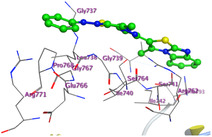	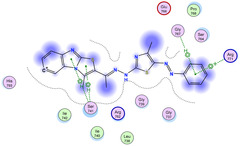
**5b**	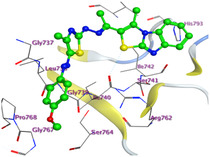	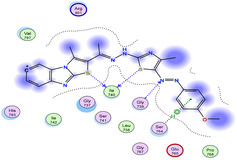
**5c**	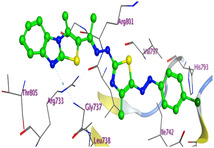	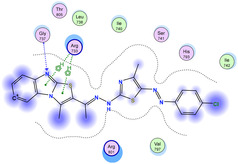
**10a**	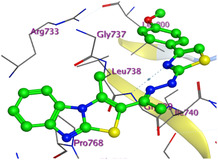	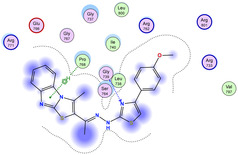
**10b**	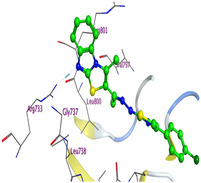	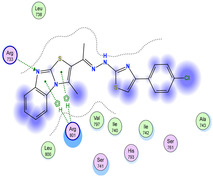
**10c**	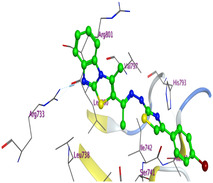	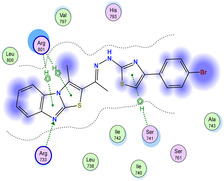
**10d**	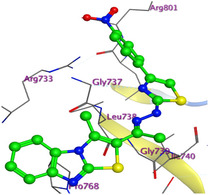	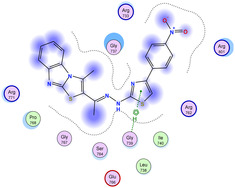
**12**	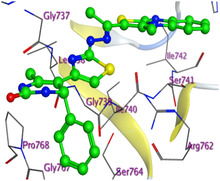	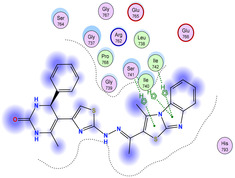
**14**	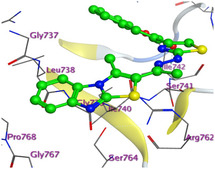	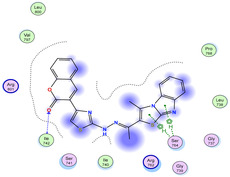
**16a**	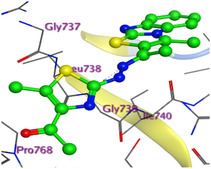	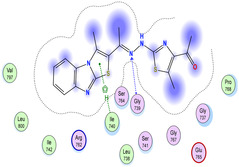
**16b**	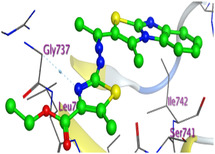	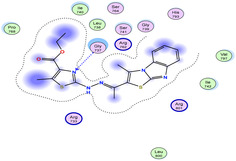
**Dox**.	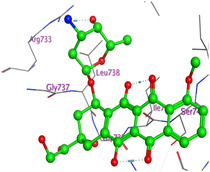	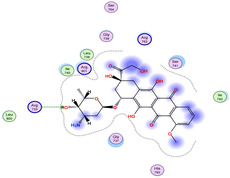

### 2.5 In Silico Pharmacokinetic Profile (ADMET)

The tested compounds (**3, 5a‐c, 10a‐d, 12, 14,** and **16a, b**) demonstrated an average human intestinal absorption ranging from 85.39% to 100%, suggesting excellent bioavailability. The volume of distribution (log L/kg) for these compounds was observed between −0.25 and 0.41, indicating moderate tissue distribution.

In terms of metabolism, most of the tested compounds (**5a‐c, 10a‐d, 12, 14,** and **16a, b**) were found to be metabolized by CYP3A4 substrates and acted as inhibitors for CYP3A4, CYP2C19, and CYP2C9 enzymes. However, compound **3** was distinct, as it was metabolized exclusively by CYP1A2 inhibitors. Additionally, derivatives **5a, 10a, 10d, 14, 16a,** and **16b** exhibited inhibitory activity against CYP1A2, while compounds **10b** and **10c** could also inhibit CYP2D6. The excretion profile of these compounds, assessed through the log values of ml/min/kg, ranged from 0.48 to 0.77, indicating their efficient elimination from the body. Finally, toxicity predictions revealed that none of the tested compounds exhibited skin sensitization toxicity, underscoring their safety profile. The detailed pharmacokinetic and toxicity data are summarized in **Table** **4**.

**Table 4:** Results of ADMET characteristics of synthetic compounds 3, 5a‐c, 10a‐d, 12, 14, 16a, b, and doxorubicin.

Cpd. NO.	Intestinal absorption numeric (% absorbed)	Distribution VDss [Log L/kg]	Metabolism	Excretion [Log ml/min/kg]	Toxicity
					Skin sensitization (categorical (yes/no)
**3**	88.28	0.27	CYP1A2 Inhibitor	0.54	No
**5a**	91.94	0.41	CYP3A4 Substrate; CYP1A2, CYP2C19, CYP2C9, and CYP3A4 inhibitor	0.50	
**5b**	93.01	0.31	CYP3A4 Substrate; CYP2C19, CYP2C9, and CYP3A4 inhibitor	0.52	
**5c**	90.56	0.40		0.49	
**10a**	87.93	−0.19	CYP3A4 Substrate; CYP1A2, CYP2C19, CYP2C9, and CYP3A4 inhibitor	0.77	
**10b**	85.45	−0.08	CYP3A4 Substrate; CYP1A2, CYP2C19, CYP2C9, CYP2D6, and CYP3A4 inhibitor	0.69	
**10c**	85.39	−0.08		0.67	
**10d**	100	−0.12	CYP3A4 Substrate; CYP1A2, CYP2C19, CYP2C9, and CYP3A4 inhibitor	0.48	
**12**	100	0.09	CYP3A4 Substrate; CYP2C19, CYP2C9, and CYP3A4 inhibitor	0.59	
**14**	89.82	−0.25	CYP3A4 Substrate; CYP1A2, CYP2C19, CYP2C9, and CYP3A4 inhibitor	0.58	
**16a**	87.54	0.00		0.56	
**16b**	87.65	−0.04		0.60	
Doxorubicin	62.10	1.71	–	0.98	

## 3 Experimental Section

### 3.1 Instruments and Materials

Elemental analysis (CHN) was carried out using a Perkin‐Elmer 2400 instrument. Thin‐layer chromatography (TLC) was performed on Merck silica gel GF254 plates, and the melting points of the products were measured using an Electrothermal Gallenkamp apparatus. The ^1^H‐NMR and ^13^C‐NMR spectra were recorded on a Jeol‐500 spectrometer, operating at 500 MHz for ^1^H‐NMR and 125 MHz for ^13^C‐NMR. Infrared (IR) spectra were obtained using potassium bromide discs on a Pye‐Unicam SP300 spectrometer. Mass spectrometric analysis was conducted using either a Thermo Scientific GC/MS ISQ model or an Agilent LC‐MSD IQ Infinity II 1260 system. Hydrazonoyl halides^[^ ^57^, ^58^, ^59^ ^]^ and 2‐bromo‐1‐arylethan‐1‐ones^[^ ^60^ ^]^ were prepared according to established procedures previously described in the literature.

### 3.2 Chemistry

#### 3.2.1 General Procedure for Reactions of Thiosemicarbazone with Hydrazonoyl Halides and α‐Haloketones

A mixture of 2‐(1‐(3‐methylbenzo[4,5]imidazo[2,1‐b]thiazol‐2‐yl)ethylidene)hydrazine‐1‐ carbothioamide (**3**) (0.303 g, 1 mmol) and 2‐oxo‐N‐arylpropanehydrazonoyl halides (**3a‐c**),^[^ ^57^, ^58^, ^59^ ^]^ N‐arylbenzohydrazonoyl chlorides (**6a,b**), 2‐bromo‐1‐arylethan‐1‐ones (**9a‐d**,^[^ ^60^ ^]^ **11, 13**), 3‐chloro‐2,4‐pentanedione (**15a**), or ethyl 2‐chloro‐3‐oxobutanoate (**15b**) (1 mmol each) in 20 mL ethanol (EtOH) was prepared, with a catalytic amount of triethylamine (TEA) added. The reaction mixture was heated for 4–6 h, and its progress was monitored using thin‐layer chromatography (TLC) with an eluent system of ethyl acetate:n‐hexane (1:1). After completion, the excess solvent was evaporated under reduced pressure, and the resulting precipitate was collected by filtration and then purified by crystallization from suitable solvents to give pure products **(5a‐d, 8a,b, 10a‐d, 12, 14**, and **16a,b**).

#### 3.2.2 3‐Methyl‐2‐(1‐(2‐(4‐methyl‐5‐(phenyldiazenyl)thiazol‐2‐yl)hydrazineylidene)ethyl)benzo[4,5]imidazo[2,1‐b]thiazole (5a)

Red solid; yield 78%, m.p: 213‐215 °C (EtOH); IR, *υ* = 3318 (NH), 3046, 2929 (C‐H), 1602 (C=N) cm^−1^; ^1^H NMR (DMSO‐d_6_, 500 MHz) *δ* = 2.33 (s, 3H, CH_3_), 2.37 (s, 3H, CH_3_), 2.47 (s, 3H, CH_3_), 6.95–7.94 (m, 9H, Ar‐H), 10.68 (s, 1H, NH) ppm; ^13^C NMR (DMSO, 125 MHz) *δ* = 9.3, 11.3, 14.5 (CH_3_), 105.2, 112.0, 119.4, 121.7, 122.0, 123.3, 126.1, 128.7, 129.1, 129.5, 129.9, 132.0, 135.4, 145.2, 147.8, 153.0, 159.5 (Ar–C and C=N) ppm; MS, *m/z* (%) 445 (M^+^, 62), 77 (100). Anal. calcd for C_22_H_19_N_7_S_2_ (445.56): C, 59.31; H, 4.30; N, 22.01. Found: C, 59.25; H, 4.16; N, 21.92%.

#### 3.2.3 2‐(1‐(2‐(5‐((4‐Methoxyphenyl)diazenyl)‐4‐methylthiazol‐2‐yl)hydrazineylidene)ethyl)‐3‐methylbenzo[4,5]imidazo[2,1‐b]thiazole (5b)

Brown solid; yield 75%, m.p: 188–190 °C (EtOH); IR, *υ* = 3340 (NH), 3053, 2927 (C‐H), 1600 (C=N) cm^−1^; ^1^H NMR (DMSO‐d_6_, 500 MHz) *δ* = 2.39 (s, 3H, CH_3_), 2.47 (s, 3H, CH_3_), 2.53 (s, 3H, CH_3_), 3.79 (s, 3H, OCH_3_), 7.21–7.95 (m, 8H, Ar‐H), 10.61 (s, 1H, NH) ppm; ^13^C NMR (DMSO, 125 MHz) *δ* = 9.0, 11.4, 14.8 (CH_3_), 56.1 (OCH_3_), 104.8, 112.3, 118.9, 121.3, 122.7, 123.7, 125.9, 129.0, 129.2, 129.6, 129.9, 132.2, 137.3, 146.0, 148.2, 154.4, 159.7 (Ar–C and C=N) ppm; MS, *m/z* (%) 475 (M^+^, 37), 105 (100). Anal. calcd for C_23_H_21_N_7_OS_2_ (475.59): C, 58.09; H, 4.45; N, 20.62. Found: C, 58.01; H, 4.33; N, 20.51%.

#### 3.2.4 2‐(1‐(2‐(5‐((4‐Chlorophenyl)diazenyl)‐4‐methylthiazol‐2‐yl)hydrazineylidene)ethyl)‐3‐methylbenzo[4,5]imidazo[2,1‐b]thiazole (5c)

Red solid; yield 82%, m.p: 229–231 °C (DMF\EtOH); IR, *υ* = 3361 (NH), 3059, 2932 (C‐H), 1607 (C=N) cm^−1^; ^1^H NMR (DMSO‐d_6_, 500 MHz) *δ* = 2.39 (s, 3H, CH_3_), 2.46 (s, 3H, CH_3_), 2.57 (s, 3H, CH_3_), 7.27–7.98 (m, 8H, Ar‐H), 10.61 (s, 1H, NH) ppm; MS, *m/z* (%) 482 (M^+^ + 2, 24), 480 (M^+^, 70), 109 (100). Anal. calcd for C_22_H_18_ClN_7_S_2_ (480.01): C, 55.05; H, 3.78; N, 20.43. Found: C, 55.13; H, 3.69; N, 20.32%.

#### 3.2.5 2‐(1‐((3,5‐Diphenyl‐1,3,4‐thiadiazol‐2(3 H)‐ylidene)hydrazineylidene)ethyl)‐3‐methylbenzo[4,5]imidazo[2,1‐b]thiazole (8a)

Yellow solid; yield 77%, m.p: 193–195 °C (EtOH); IR, *υ* = 3052, 2923 (C‐H), 16 082 (C=N) cm^−1^; ^1^H NMR (DMSO‐d_6_, 500 MHz) *δ* = 2.38 (s, 3H, CH_3_), 2.47 (s, 3H, CH_3_), 7.07–7.92 (m, 14H, Ar‐H) ppm; ^13^C NMR (DMSO, 125 MHz) *δ* = 10.0, 12.9 (CH_3_), 113.6, 117.2, 120.4, 121.2, 123.6, 124.7, 125.3, 127.3, 129.3, 129.5, 130.1, 130.7, 132.1, 133.1, 135.8, 137.4, 144.1, 147.4, 151.7, 159.2 (Ar–C and C=N) ppm; MS, *m/z* (%) 480 (M^+^, 100), 77 (95). Anal. calcd for C_26_H_20_N_6_S_2_ (480.61): C, 64.98; H, 4.19; N, 17.49. Found: C, 65.02; H, 4.27; N, 17.58%.

#### 3.2.6 2‐(1‐((3‐(4‐Chlorophenyl)‐5‐phenyl‐1,3,4‐thiadiazol‐2(3 H)‐ylidene)hydrazineylidene)ethyl)‐3‐methylbenzo[4,5]imidazo[2,1‐b]thiazole (8b)

Yellow solid; yield 79%, m.p: 213–215 °C (EtOH); IR, *υ* = 3063, 29238 (C‐H), 1612 (C=N) cm^−1^; ^1^H NMR (DMSO‐d_6_, 500 MHz) *δ* = 2.39 (s, 3H, CH_3_), 2.47 (s, 3H, CH_3_), 7.35–7.97 (m, 13H, Ar‐H) ppm; MS, *m/z* (%) 517 (M^+^+ 2, 9), 515 (M^+^, 29), 105 (100). Anal. calcd for C_26_H_19_ClN_6_S_2_, (515.05) C, 60.63; H, 3.72; Cl, 6.88; N, 16.32. Found: C, 60.72; H, 3.80; Cl, 6.97; N, 16.41.%.

#### 3.2.7 2‐(1‐(2‐(4‐(4‐Methoxyphenyl)thiazol‐2‐yl)hydrazineylidene)ethyl)‐3‐methylbenzo[4,5]imidazo[2,1‐b]thiazole (10a)

Orange solid; yield 79%, m.p: 178‐180 °C (EtOH); IR, *υ* = 3335 (NH), 3061, 2927 (C‐H), 1611 (C=N) cm^−1^; ^1^H NMR (DMSO‐d_6_, 500 MHz) *δ* = 2.27 (s, 3H, CH_3_), 2.38 (s, 3H, CH_3_), 3.79 (s, 3H, OCH_3_), 7.17–7.95 (m, 9H, Ar‐H and thiazole‐H5), 10.62 (s, 1H, NH) ppm; ^13^C NMR (DMSO, 125 MHz) *δ* = 10.6, 14.8 (CH_3_), 56.4 (OCH_3_), 107.6, 110.2, 116.2, 117.0, 118.7, 120.1, 121.5, 124.6, 131.4, 131.6, 133.7, 138.5, 138.7, 148.5, 149.3, 159.1, 163.3 (Ar–C and C=N) ppm; MS, *m/z* (%) 433 (M^+^, 29), 127 (100). Anal. calcd for C_22_H_19_N_5_OS_2_, (433.55): C, 60.95; H, 4.42; N, 16.15. Found: C, 61.05; H, 4.51; N, 16.23%.

#### 3.2.8 2‐(1‐(2‐(4‐(4‐Chlorophenyl)thiazol‐2‐yl)hydrazineylidene)ethyl)‐3‐methylbenzo[4,5]imidazo[2,1‐b]thiazole (10b)

Yellow solid; yield 80%, m.p: 241–243 °C (DMF); IR, *υ* = 3343 (NH), 3053, 2929 (C‐H), 1615 (C=N) cm^−1^; ^1^H NMR (DMSO‐d_6_, 500 MHz) *δ* = 2.40 (s, 3H, CH_3_), 2.47 (s, 3H, CH_3_), 7.13–7.92 (m, 9H, Ar‐H and thiazole‐H5), 10.63 (s, 1H, NH) ppm; MS, *m/z* (%) 439 (M^+^ + 2, 15), 437 (M^+^, 47), 173 (100). Anal. calcd for C_21_H_16_ClN_5_S_2_ (437.96): C, 57.59; H, 3.68; Cl, 8.09; N, 15.99. Found: C, 57.67; H, 3.77; Cl, 8.18; N, 16.08%.

#### 3.2.9 2‐(1‐(2‐(4‐(4‐Bromophenyl)thiazol‐2‐yl)hydrazineylidene)ethyl)‐3‐methylbenzo[4,5]imidazo[2,1‐b]thiazole (10c)

Yellow solid; yield 81%, m.p: 203–205 °C (EtOH); IR, *υ* = 3350 (NH), 3062, 2928 (C‐H), 1615 (C=N) cm^−1^; ^1^H NMR (DMSO‐d_6_, 500 MHz) *δ* = 2.28 (s, 3H, CH_3_), 2.45 (s, 3H, CH_3_), 7.12–7.95 (m, 9H, Ar‐H and thiazole‐H5), 10.61 (s, 1H, NH) ppm; MS, *m/z* (%) 484 (M^+^ + 2, 47), 482 (M^+^, 52), 163 (100). Anal. calcd for C_21_H_16_BrN_5_S_2_ (482.42): C, 52.28; H, 3.34; Br, 16.56; N, 14.52. Found: C, 52.36; H, 3.45; Br, 16.67; N, 14.60%.

#### 3.2.10 3‐Methyl‐2‐(1‐(2‐(4‐(4‐nitrophenyl)thiazol‐2‐yl)hydrazineylidene)ethyl)benzo[4,5]imidazo[2,1‐b]thiazole (10d)

Brown solid; yield 82%, m.p: 255–257 °C (DMF); IR, *υ* = 3372 (NH), 3067, 2934 (C‐H), 1612 (C=N) cm^−1^; ^1^H NMR (DMSO‐d_6_, 500 MHz) *δ* = 2.41 (s, 3H, CH_3_), 2.47 (s, 3H, CH_3_), 7.38–8.26 (m, 9H, Ar‐H and thiazole‐H5), 10.81 (s, 1H, NH) ppm; MS, *m/z* (%) 448 (M^+^, 82), 137 (100). Anal. calcd for C_21_H_16_N_6_O_2_S_2_ (448.52): C, 56.24; H, 3.60; N, 18.74;. Found: C, 56.32; H, 3.71; N, 18.81%.

#### 3.2.11 6‐Methyl‐5‐(2‐(2‐(1‐(3‐methylbenzo[4,5]imidazo[2,1‐b]thiazol‐2‐yl)ethylidene)hydrazineyl)thiazol‐4‐yl)‐4‐phenyl‐3,4‐dihydropyrimidin‐2(1H)‐one (12)

Brown solid; yield 71%, m.p: 243–245 °C (DMF); IR, *υ* = 3407, 3326, 3241 (3NH), 3045, 2925 (C‐H), 1673 (C=O), 1603 (C=N) cm^−1^; ^1^H NMR (DMSO‐d_6_, 500 MHz) *δ* = 2.27 (s, 3H, CH_3_), 2.39 (s, 3H, CH_3_), 2.47 (s, 3H, CH_3_), 5.71 (s, 1H, CH), 7.14–7.97 (m, 11H, Ar‐H, thiazole‐H5, and NH), 9.88 (s, 1H, NH), 10.67 (s, 1H, NH) ppm; MS, *m/z* (%) 513 (M^+^, 25), 194 (100). Anal. calcd for C_26_H_23_N_7_OS_2_ (513.64): C, 60.80; H, 4.51; N, 19.09. Found: C, 60.91; H, 4.60; N, 19.18%.

#### 3.2.12 3‐(2‐(2‐(1‐(3‐Methylbenzo[4,5]imidazo[2,1‐b]thiazol‐2‐yl)ethylidene)hydrazineyl)thiazol‐4‐yl)‐2H‐chromen‐2‐one (14)

Brown solid; yield 77%, m.p: 239–241 °C (DMF); IR, *υ* = 3370 (NH), 3062, 2927 (C‐H), 1729 (C=O), 1609 (C=N) cm^−1^; ^1^H NMR (DMSO‐d_6_, 500 MHz) *δ* = 2.29 (s, 3H, CH_3_), 2.45 (s, 3H, CH_3_), 7.18–7.93 (m, 9H, Ar‐H and thiazole‐H5), 8.23 (s, 1H, coumarin‐H4), 10.65 (s, 1H, NH) ppm; MS, *m/z* (%) 471 (M^+^, 57), 142 (100). Anal. calcd for C_24_H_17_N_5_O_2_S_2_ (471.55): C, 61.13; H, 3.63; N, 14.85. Found: C, 61.22; H, 3.71; N, 14.91%.

#### 3.2.13 1‐(5‐Methyl‐2‐(2‐(1‐(3‐methylbenzo[4,5]imidazo[2,1‐b]thiazol‐2‐yl)ethylidene)hydrazineyl)thiazol‐4‐yl)ethan‐1‐one (16a)

Yellow solid; yield 78%, m.p: 205–207 °C (EtOH); IR, *υ* = 3353 (NH), 3047, 2924 (C‐H), 1704 (C=O), 1607 (C=N) cm^−1^; ^1^H NMR (DMSO‐d_6_, 500 MHz) *δ* = 2.27 (s, 3H, CH_3_), 2.35 (s, 3H, CH_3_), 2.47 (s, 3H, CH_3_), 2.57 (s, 3H, CH_3_), 7.25–7.71 (m, 4H, Ar‐H), 10.69 (s, 1H, NH) ppm; MS, *m/z* (%) 383 (M^+^, 33), 118 (100). Anal. calcd for C_18_H_17_N_5_OS_2_ (383.49): C, 56.38; H, 4.47; N, 18.26. Found: C, 56.38; H, 4.47; N, 18.26%.

#### 3.2.14 1‐(5‐Methyl‐2‐(2‐(1‐(3‐methylbenzo[4,5]imidazo[2,1‐b]thiazol‐2‐yl)ethylidene)hydrazineyl)thiazol‐4‐yl)ethan‐1‐one (16b)

Yellow solid; yield 74%, m.p: 183–185 °C (EtOH); IR, *υ* = 3347 (NH), 3052, 2925 (C‐H), 1723 (C=O), 1609 (C=N) cm^−1^; ^1^H NMR (DMSO‐d_6_, 500 MHz) *δ* = 1.37 (t, 3H, CH_3_), 2.28 (s, 3H, CH_3_), 2.45 (s, 3H, CH_3_), 4.27 (q, 2H, CH_2_), 7.28‐7.67 (m, 4H, Ar‐H), 10.66 (s, 1H, NH) ppm; ^13^C NMR (DMSO, 125 MHz) *δ* = 11.0, 13.3, 13.8, 16.0 (CH_3_), 59.6 (CH_2_), 121.2, 122.7, 123.7, 125.9, 129.0, 129.2, 129.6, 129.9, 132.2, 137.3, 142.2, 148.2, 157.4 (Ar–C and C=N), 168.8 (C=O) ppm; MS, *m/z* (%) 413 (M^+^, 72), 139 (100). Anal. calcd for C_19_H_19_N_5_O_2_S_2_ (413.51): C, 55.19; H, 4.63; N, 16.94. Found: C, 55.19; H, 4.63; N, 16.94%.

### 3.3 Cytotoxicity Assay

To thoroughly evaluate cytotoxicity and antitumor activity, cell lines were seeded into 96‐well plates at a density of 5 × 10^4^ cells per well in 100 μL of growth medium. After allowing the cells to adhere and grow for 24 h, the test compounds were added at eight serially diluted concentrations to each well, ensuring a comprehensive dose‐response analysis. Each concentration was tested in six replicates to ensure statistical reliability and reproducibility of the results. Control wells were included to assess baseline cell viability, with one set containing only the growth medium and another containing 0.5% DMSO, the maximum concentration of solvent used to dissolve the compounds, ensuring it did not interfere with the experiment.

Following an additional 24 h of incubation with the compounds, the cytotoxic effects were measured using the 3‐(4,5‐Dimethylthiazol‐2‐yl)‐2,5‐diphenyltetrazolium bromide (MTT) assay, a standard colorimetric method for assessing cell metabolic activity as an indicator of cell viability, proliferation, and cytotoxicity. MTT reduction by mitochondrial dehydrogenase to formazan crystals, correlating with viable cell count, was quantified by measuring absorbance at 570 nm using a microplate reader. The results were used to determine IC_50_ values for each compound, offering clearer insight into their potency in inhibiting cell growth.^[^ ^61^, ^62^ ^]^

### 3.4 Docking Study

Compounds exhibiting the lowest MIC values from the MTT assay were chosen for docking studies with the target protein. The selected molecules were sketched using ChemDraw Professional 16.0, and their molecular modeling was carried out using MOE software. Energy minimization was performed using the Merck Molecular Force Field 94× (MMFF94×) until the RMSD gradient reached 0.1 kcal mol^−1^ Å^−1^.^[^ ^63^ ^]^ The optimized structures were then saved in MDB format for subsequent docking simulations.

The X‐ray crystal structure of the target enzyme (PDB ID: 6MTU, resolution: 2.14 Å) was obtained from the RCSB Protein Data Bank (RCSB PDB).^[^ ^64^, ^65^ ^]^ Protein preparation included the following steps: (1) retaining the SEP co‐crystal ligand while removing water molecules, (2) adding hydrogen atoms and fixing broken bonds, (3) generating dummy atoms for broad site searches using Alpha Site Finder, (4) identifying and defining the binding pocket, and (5) saving the processed structure as an MOE file for ligand docking studies. The prepared protein structure is presented in Figure 2, and the resulting ligand interactions with amino acid residues in the active site were thoroughly analyzed to elucidate their binding behavior.

Molecular docking was performed using MOE software targeting the ATP‐binding pocket of CDK2 (PDB ID: 6MTU). The docking center was defined around the co‐crystallized ligand within the active site, and a grid box was automatically generated to encompass the binding cavity using Alpha Site Finder, covering ≈20 × 20 × 20 Å around the key binding residues. Ligand placement was done using the Triangle Matcher method, and scoring was based on the London dG function followed by refinement with forcefield‐based energy minimization.

After docking, interactions with amino acid residues were analyzed in both 2D and 3D views. All docking procedures and scoring were performed and recorded following established protocols.^[^ ^66^ ^]^

### 3.5 In Silico Pharmacokinetic Profile (ADMET)

The pharmacokinetic profile of doxorubicin and the most significant synthesized compounds was predicted using pkCSM.^[^ ^67^ ^]^ This publicly available web server (http://structure.bioc.cam.ac.UK/pkcsm) provides a comprehensive platform for rapid evaluation of pharmacokinetic^[^ ^68^ ^]^ and toxicity properties.^[^ ^69^ ^]^ Predicting ADMET features of new compounds involves integrating their pharmacokinetic and toxicological characteristics.^[^ ^70^ ^]^

## 4 Conclusions

In summary, this study presents the design and synthesis of a novel class of benzimidazothiazole–thiazole hybrids with potent anticancer activity against the HCT‐116 colon carcinoma cell line. Among these, compound **16b** exhibited superior efficacy compared to doxorubicin, supported by consistent molecular docking and favorable ADMET profiles. These findings highlight not only the promising therapeutic potential of these hybrids as lead anticancer agents but also their relevance as multifunctional scaffolds in the broader context of medicinal chemistry. The dual validation through in vitro and in silico approaches reinforces their value for future development in cancer biology and targeted drug design.

## Conflict of Interest

The authors declarer no conflict of interest.

## Author Contributions

**Bader Huwaimel**: conceptualization (equal); funding acquisition (lead); methodology (equal); writing—original draft (equal); and writing—review and editing (equal). **Amr S. Abouzied**: formal analysis (lead); resources (equal); writing—original draft (lead); and writing—review and editing (equal). **Magdi E. A. Zaki**: data curation (equal); formal analysis (equal); project administration (equal); supervision (lead); writing—review and editing (lead). **Abdulwahab Alamri**: funding acquisition (equal); resources (equal); supervision (equal); and writing—review and editing (lead). **Basant Farag**: methodology (lead); software (lead); and writing—original draft (equal); **Saad Alqarni**: formal analysis (equal); methodology (equal); software (equal); and visualization (equal). **Sobhi M. Gomha**: conceptualization (equal); formal analysis (equal); investigation (supporting); writing—original draft (lead); and writing—review and editing (equal).

## Supporting information

Supplementary Material

## Data Availability

The data that support the findings of this study are available from the corresponding author upon reasonable request.
