# Supplementary material for: Synthesis, Anticancer Evaluation, and Molecular Docking of Novel Thiazolobenzimidazole–Thiazole Hybrids as Potent Colon Cancer Inhibitors
Source: ChemistryOpen. 2025 Jul 11;14(11):e202500288. doi: 10.1002/open.202500288 (PMC12598833; doi:10.1002/open.202500288)
Supplement: Supplementary file 1 — Supplementary Material [file OPEN-14-e202500288-s001.pdf]

Supplementary material file

Synthesis, Anticancer Evaluation, and Molecular Docking of Novel Thiazolobenzimidazole–Thiazole Hybrids as Potent Colon Cancer Inhibitors

Bader Huwaimel[1,2], Amr S. Abouzied[1,2], Magdi E. A. Zaki[3*], Abdulwahab Alamri[4], Basant Farag[5], Saad Alqarni[1,2], Sobhi M. Gomha[6*]

[1]Department of Pharmaceutical Chemistry, College of Pharmacy, University of Ha’il, Ha’il 55473, Saudi Arabia; b.huwaimel@uoh.edu.sa (BH), as.ibrahim@uoh.edu.sa (AA), s.alqarni@uoh.edu.sa (SA).

[2]Medical and Diagnostic Research Center, University of Ha'il, Hail 55473, Saudi Arabia.

[3]Department of Chemistry, Faculty of Science, Imam Mohammed Ibn Saud Islamic University (IMSIU), Riyadh 11623, Saudi Arabia, mezaki@imamu.edu.sa

[4]Department of Pharmacology and Toxicology, College of Pharmacy, University of Hail, Hail 55211, Saudi Arabia; a.alamry@uoh.edu.sa

[5]Department of Chemistry, Faculty of Science, Zagazig University, Zagazig 44519, Egypt; basantfarag@zu.edu.eg

[5]Department of Chemistry, Faculty of Science, Islamic University of Madinah, Madinah 42351, Saudi Arabia, smgomha@iu.edu.sa

* Corresponding authors: *smgomha@iu.edu.sa (SG), mezaki@imamu.edu.sa (MZ)*

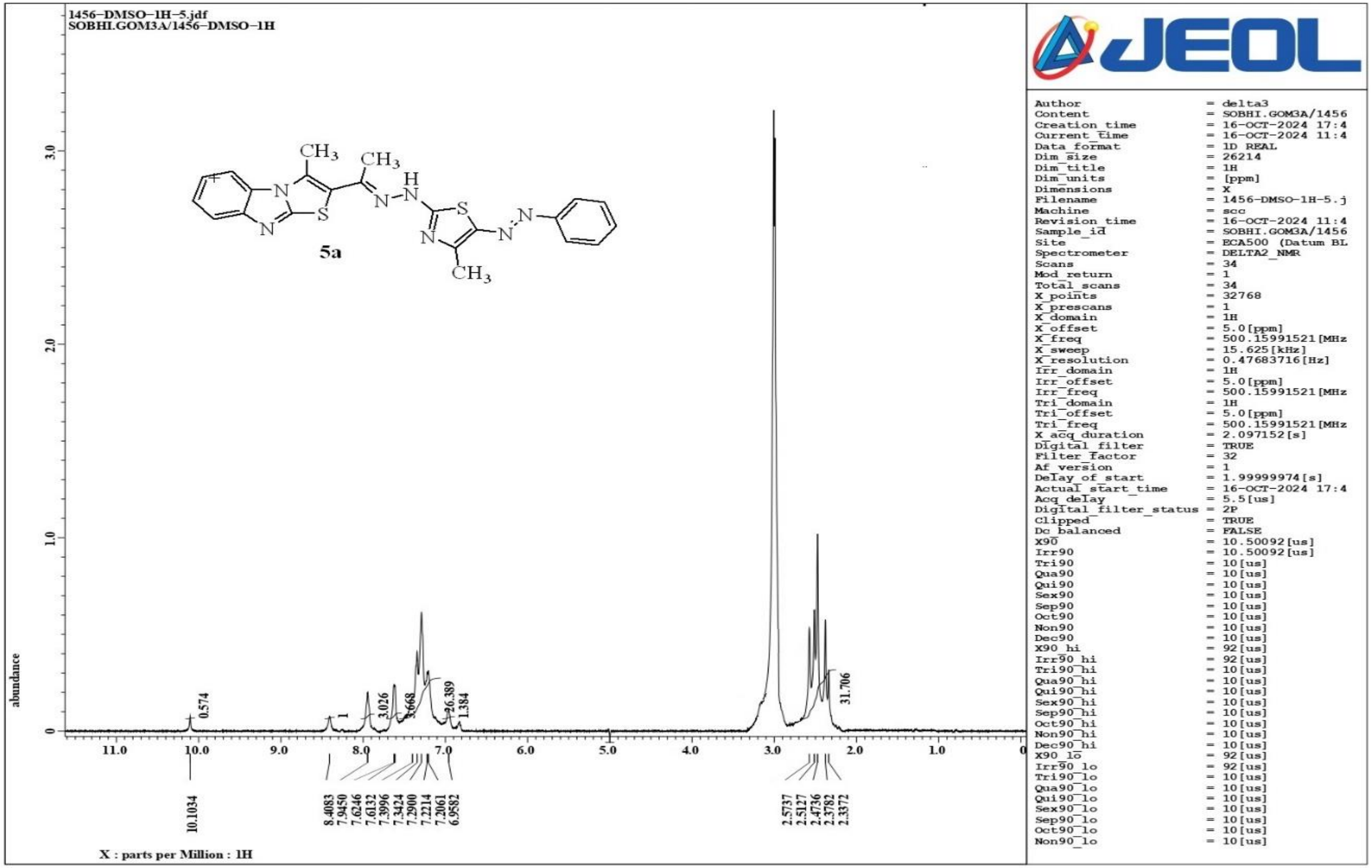

^{1}H-NMR spectra of compound **5a**

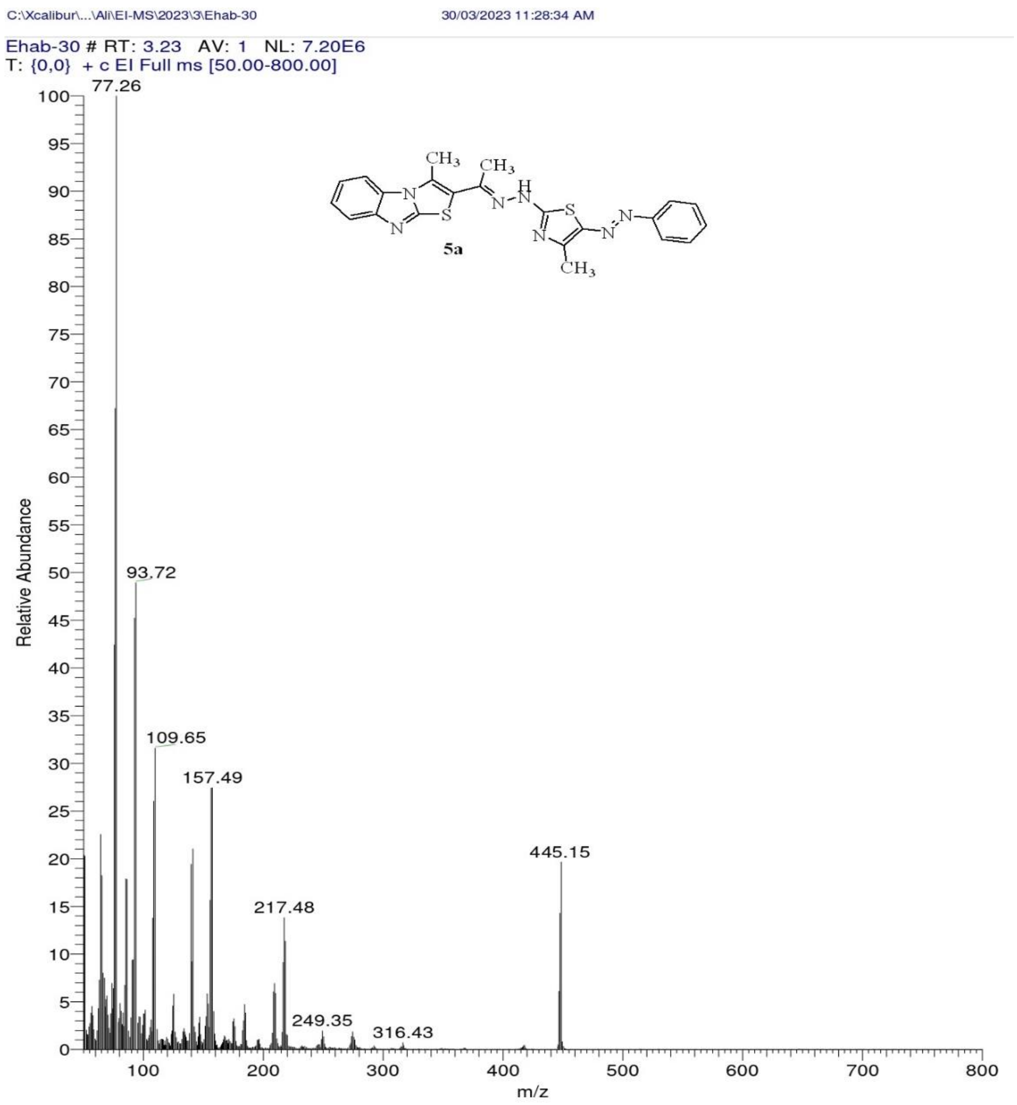

Mass spectra of compound **5a**

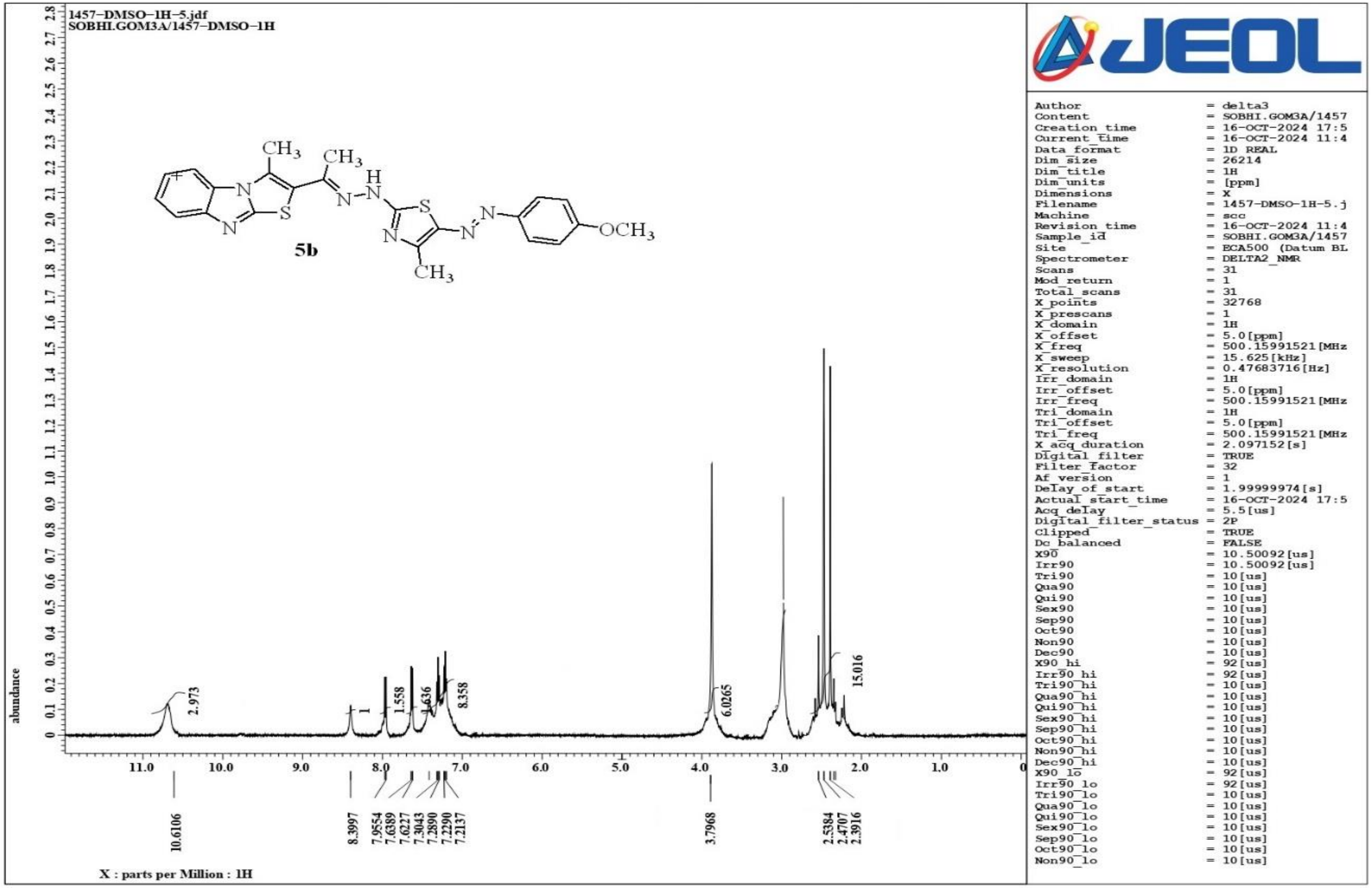

^{1}H-NMR spectra of compound **5b**

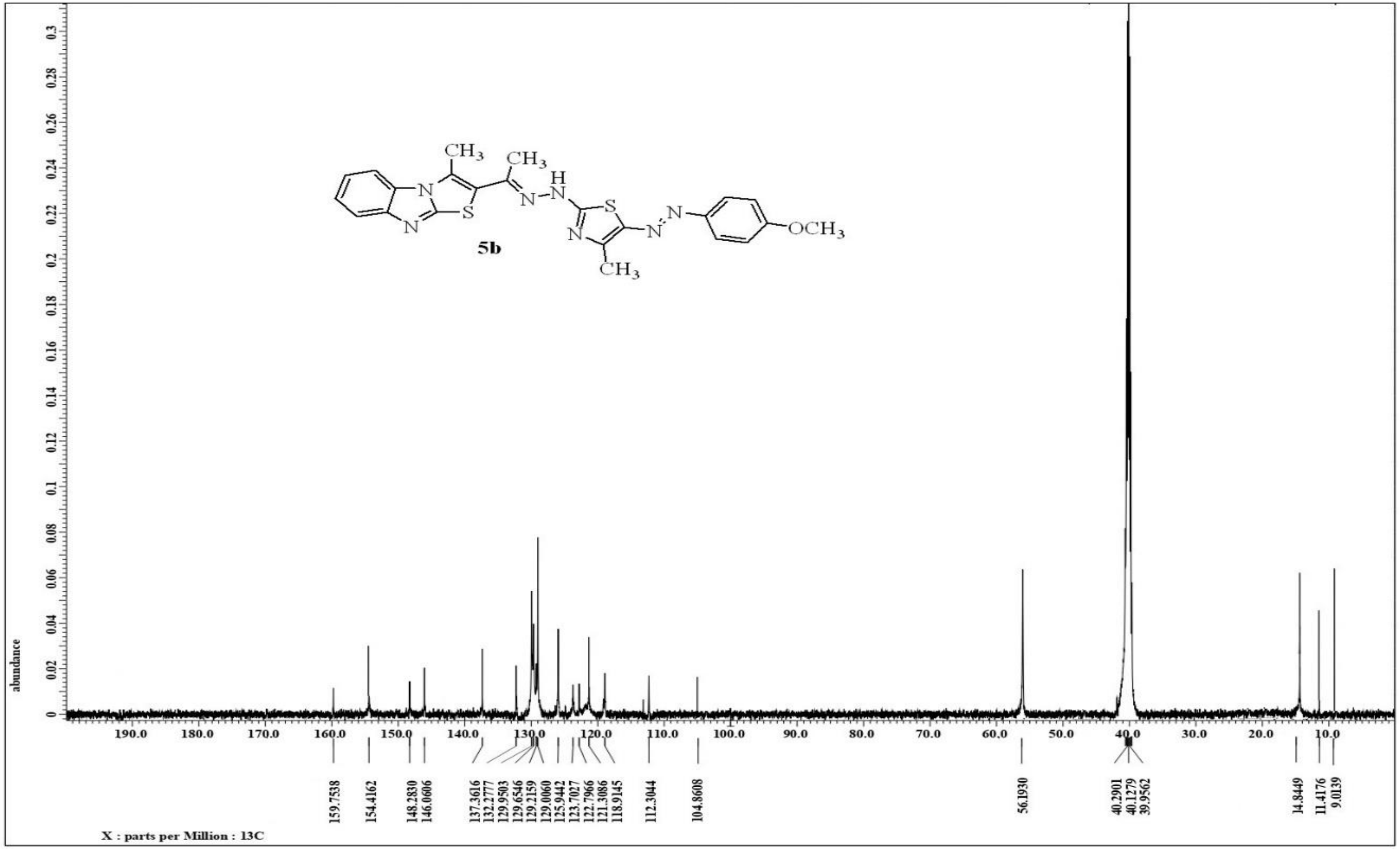

^{13}C-NMR spectra of compound **5b**

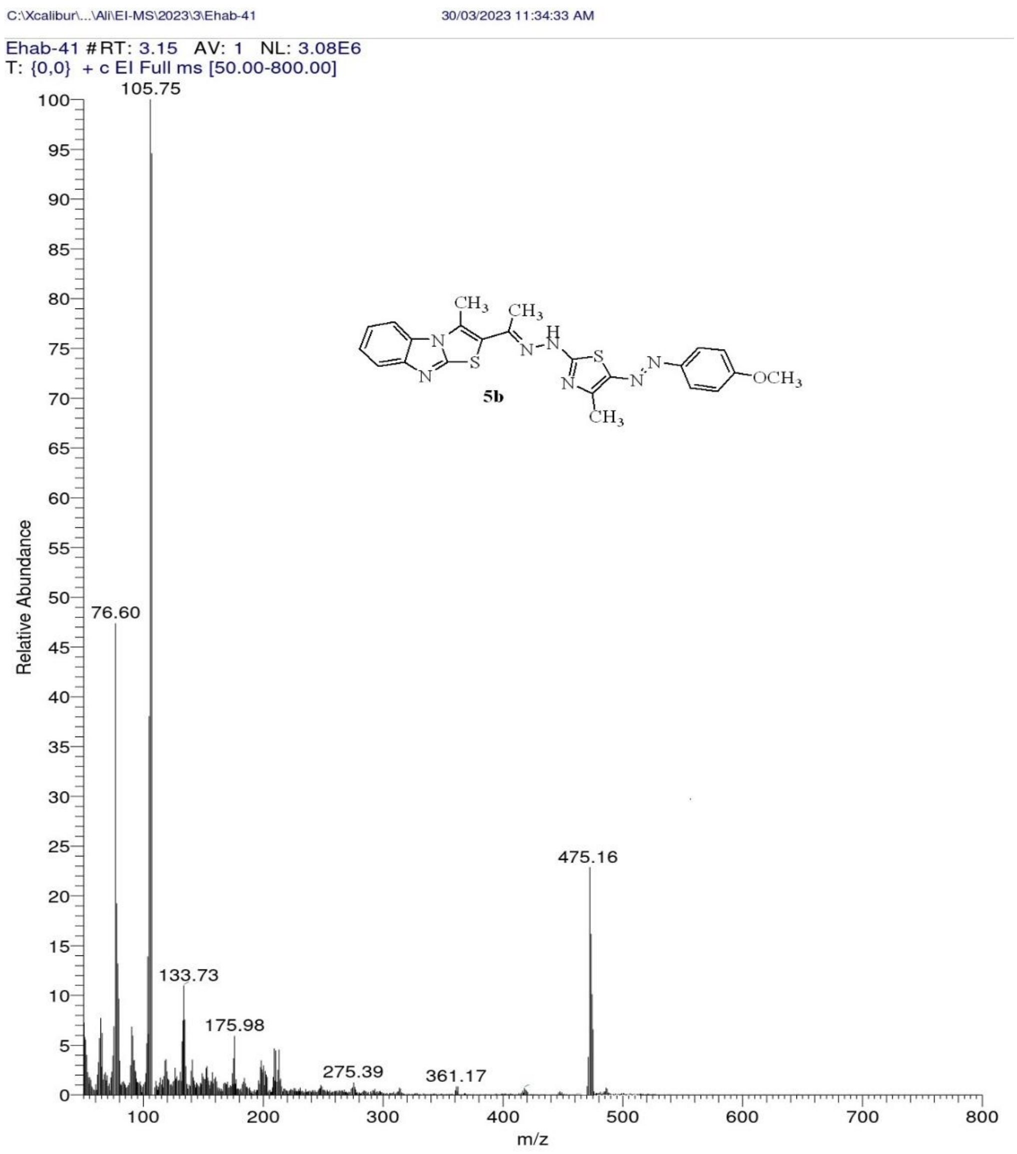

Mass spectra of compound **5b**

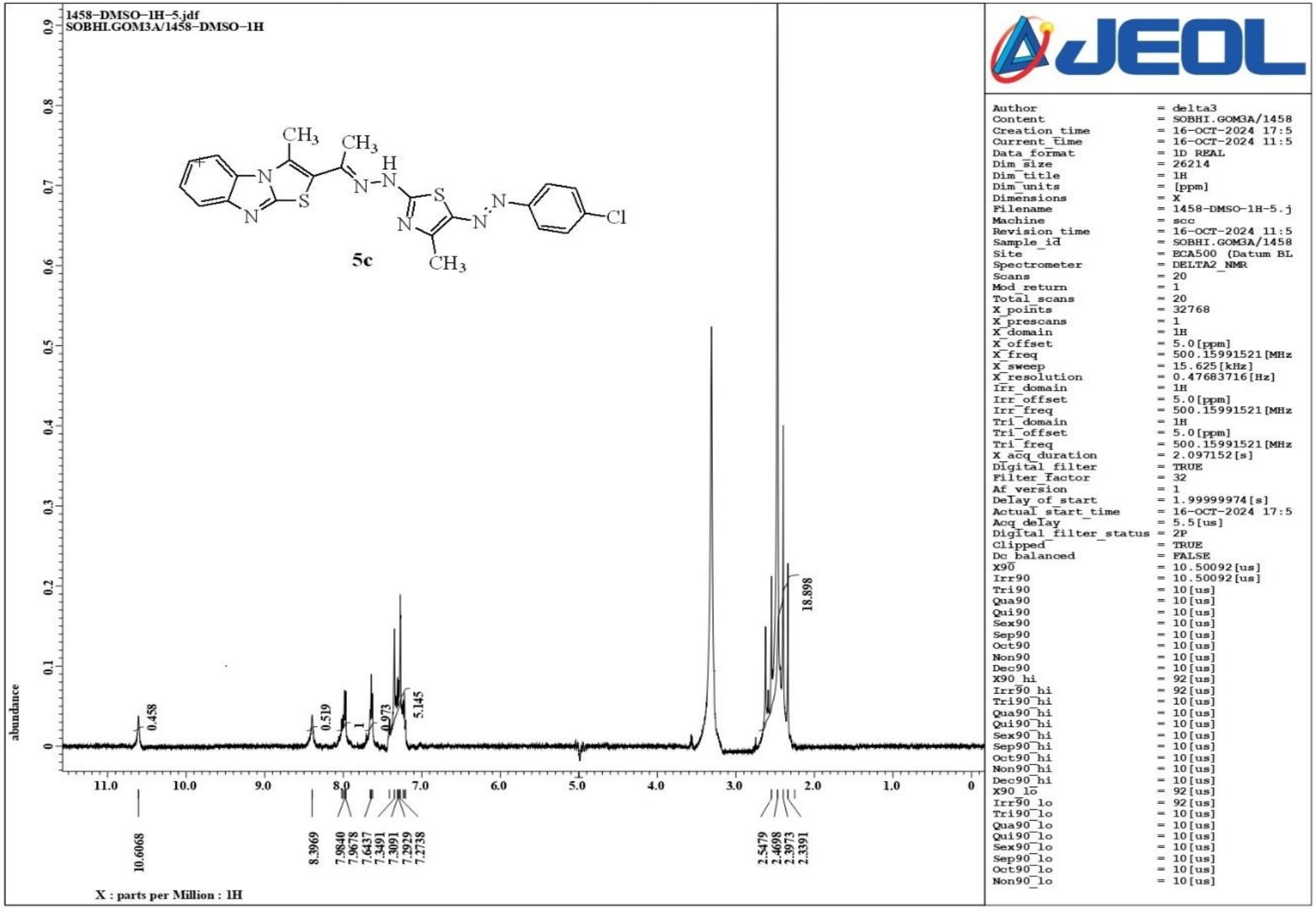

^{1}H-NMR spectra of compound **5c**

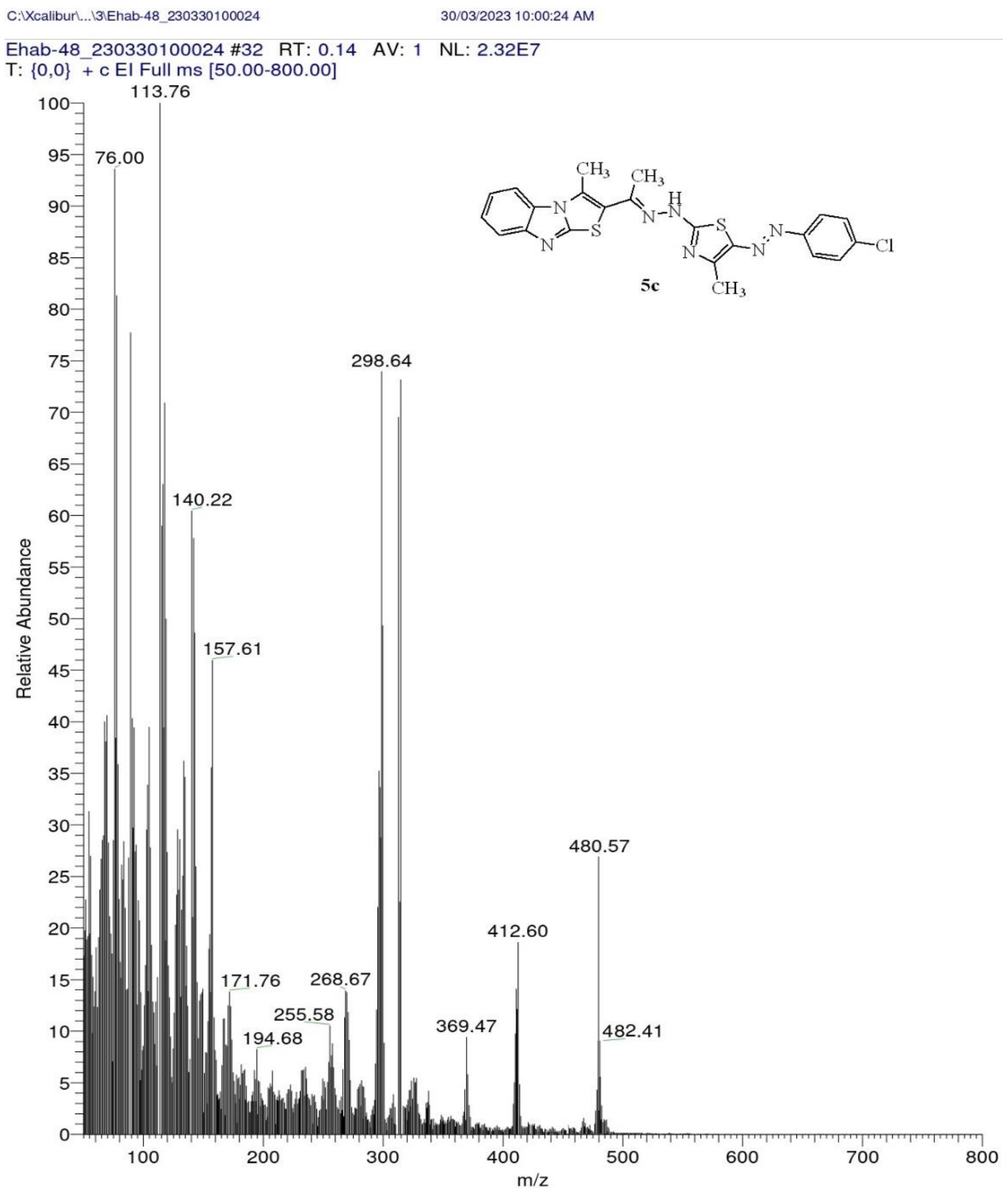

Mass spectra of compound **5c**

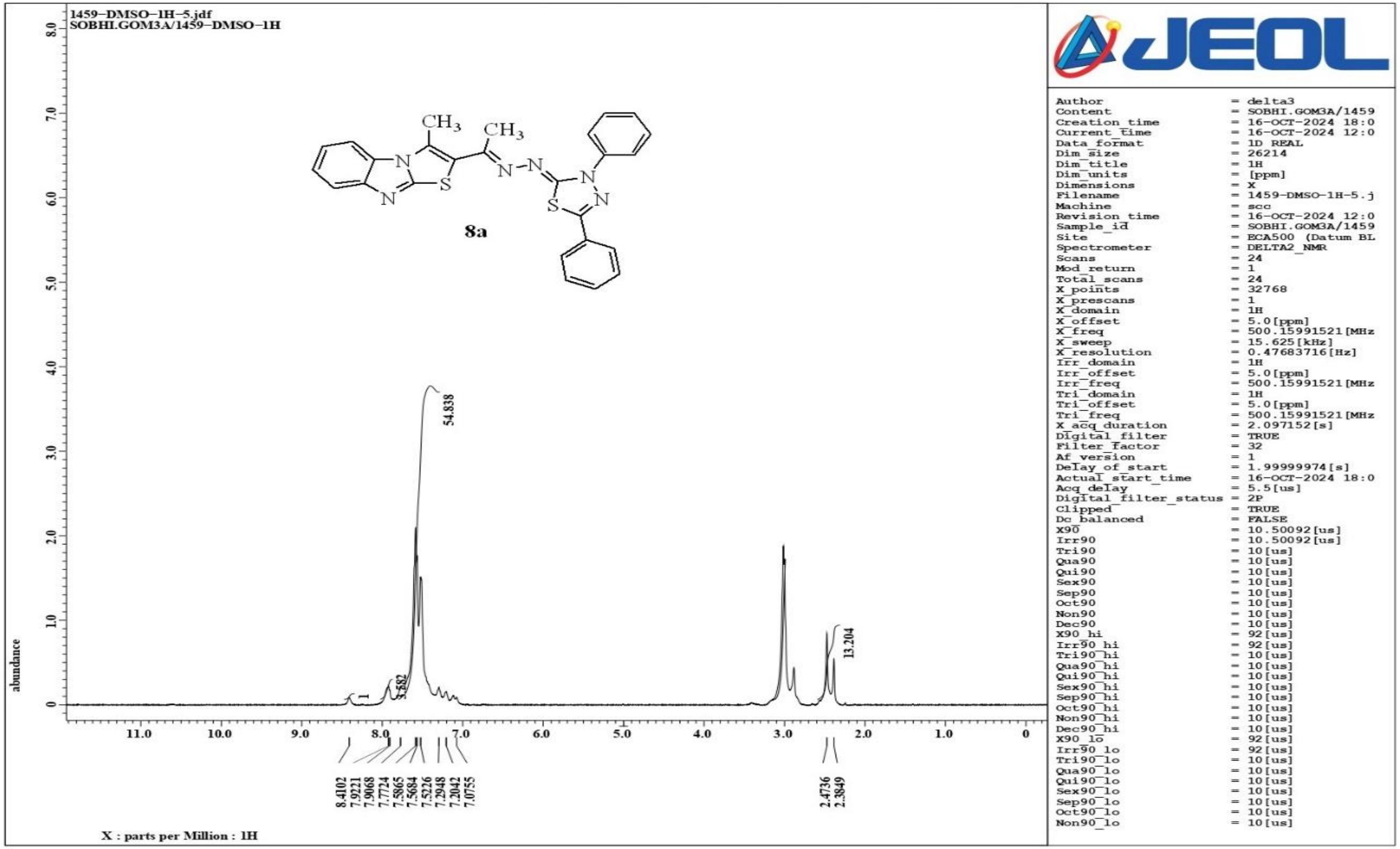

^{1}H-NMR spectra of compound **8a**

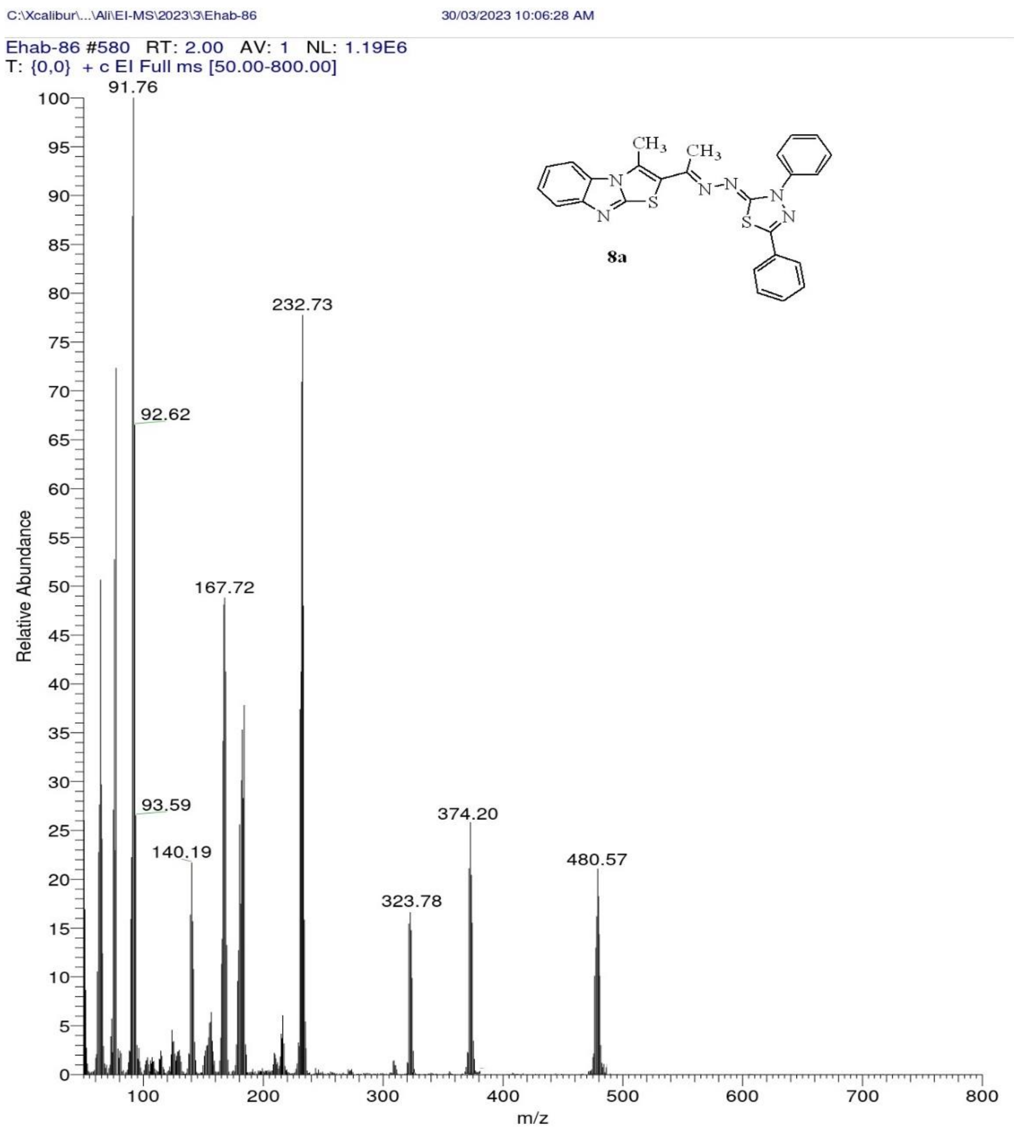

Mass spectra of compound **8a**

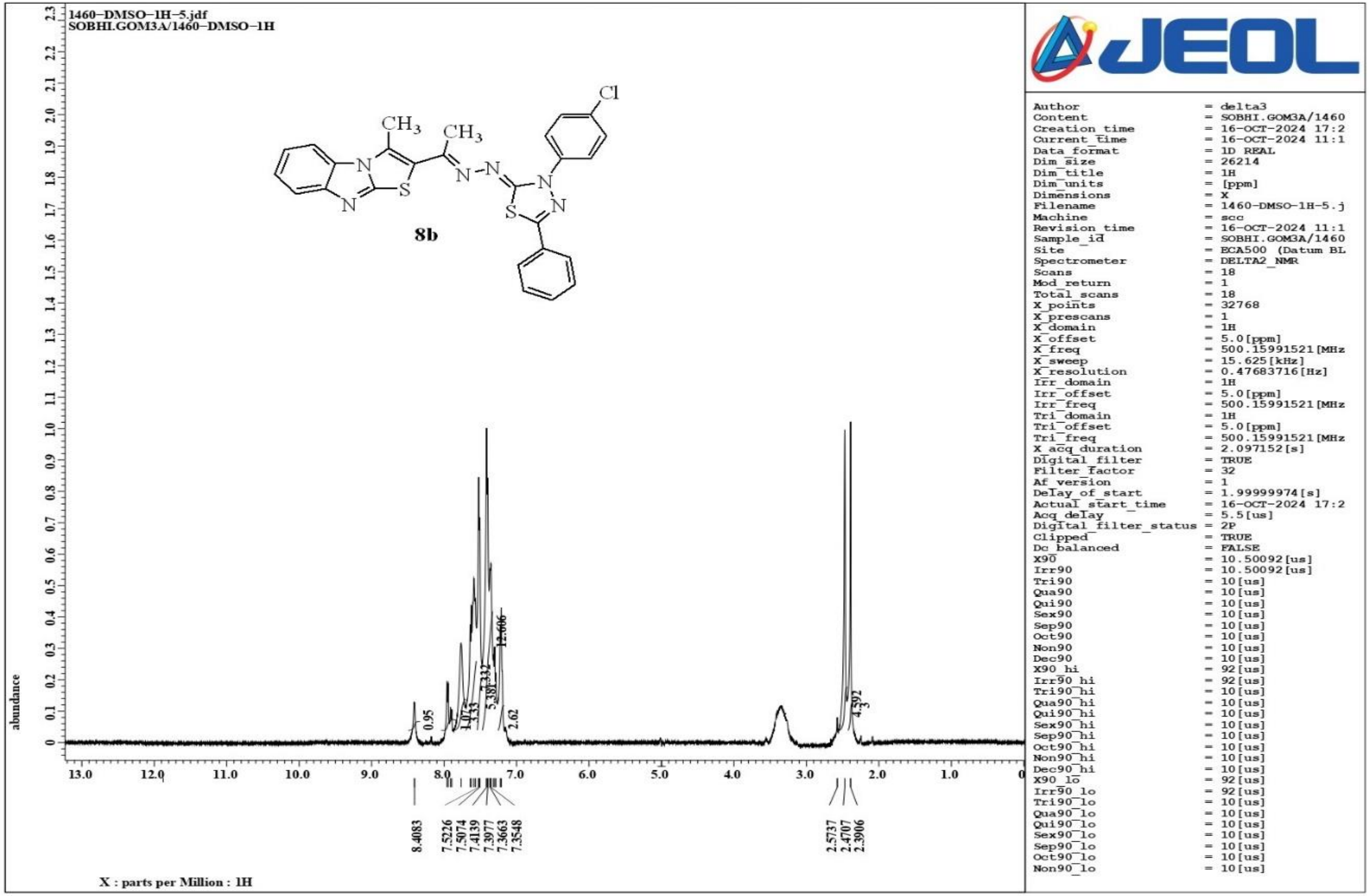

^{1}H-NMR spectra of compound **8b**

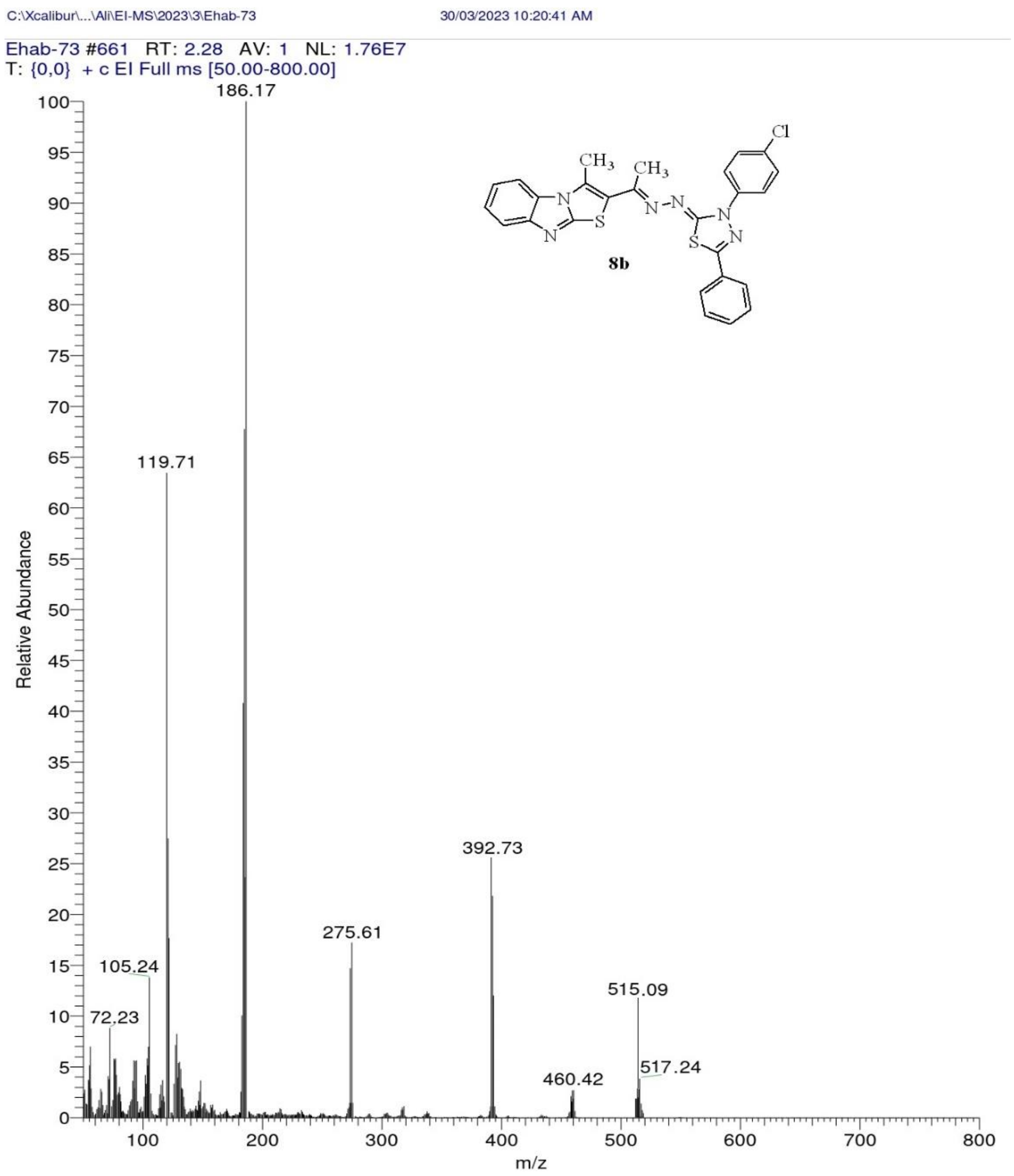

Mass spectra of compound **8b**

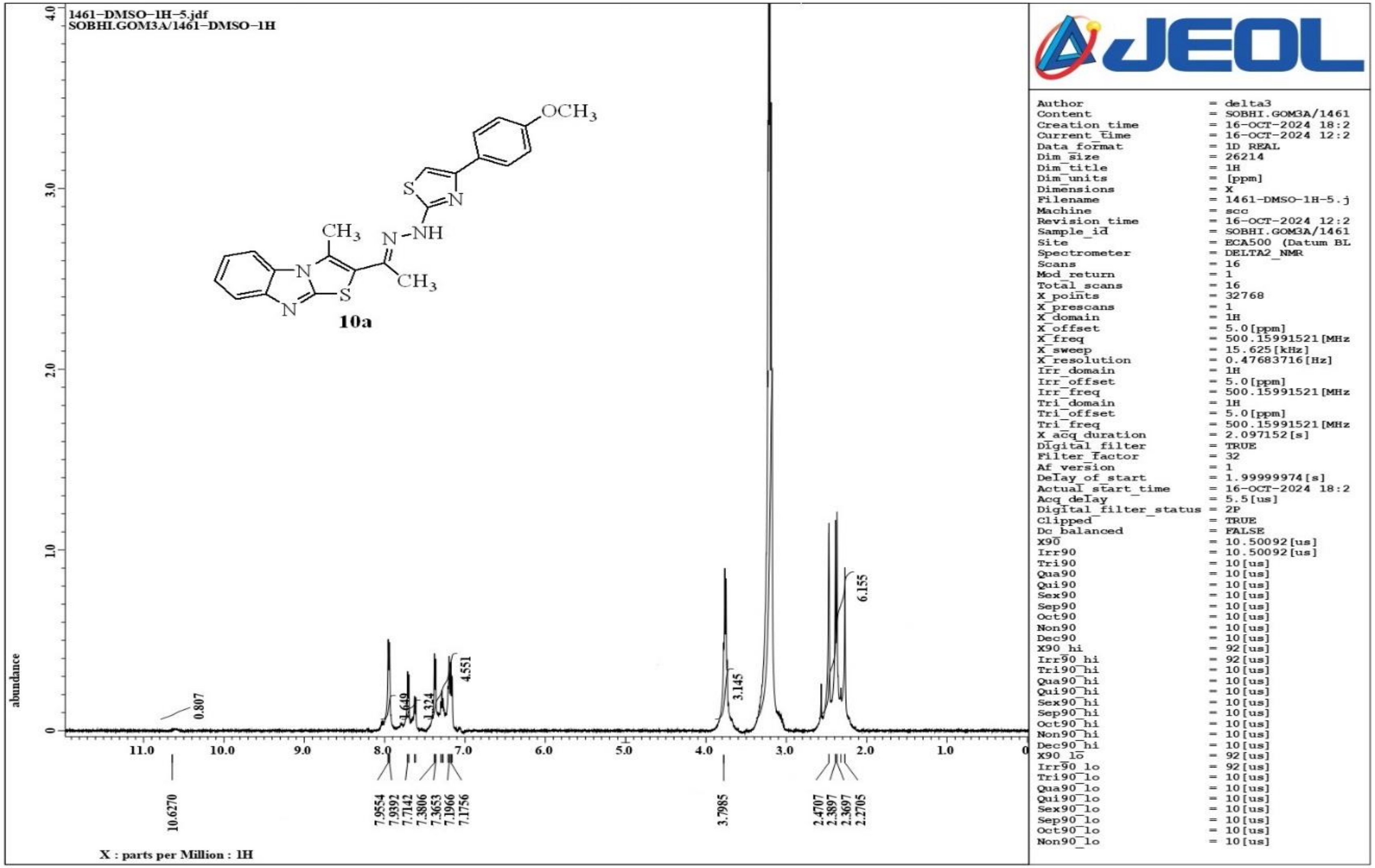

^{1}H-NMR spectra of compound **10a**

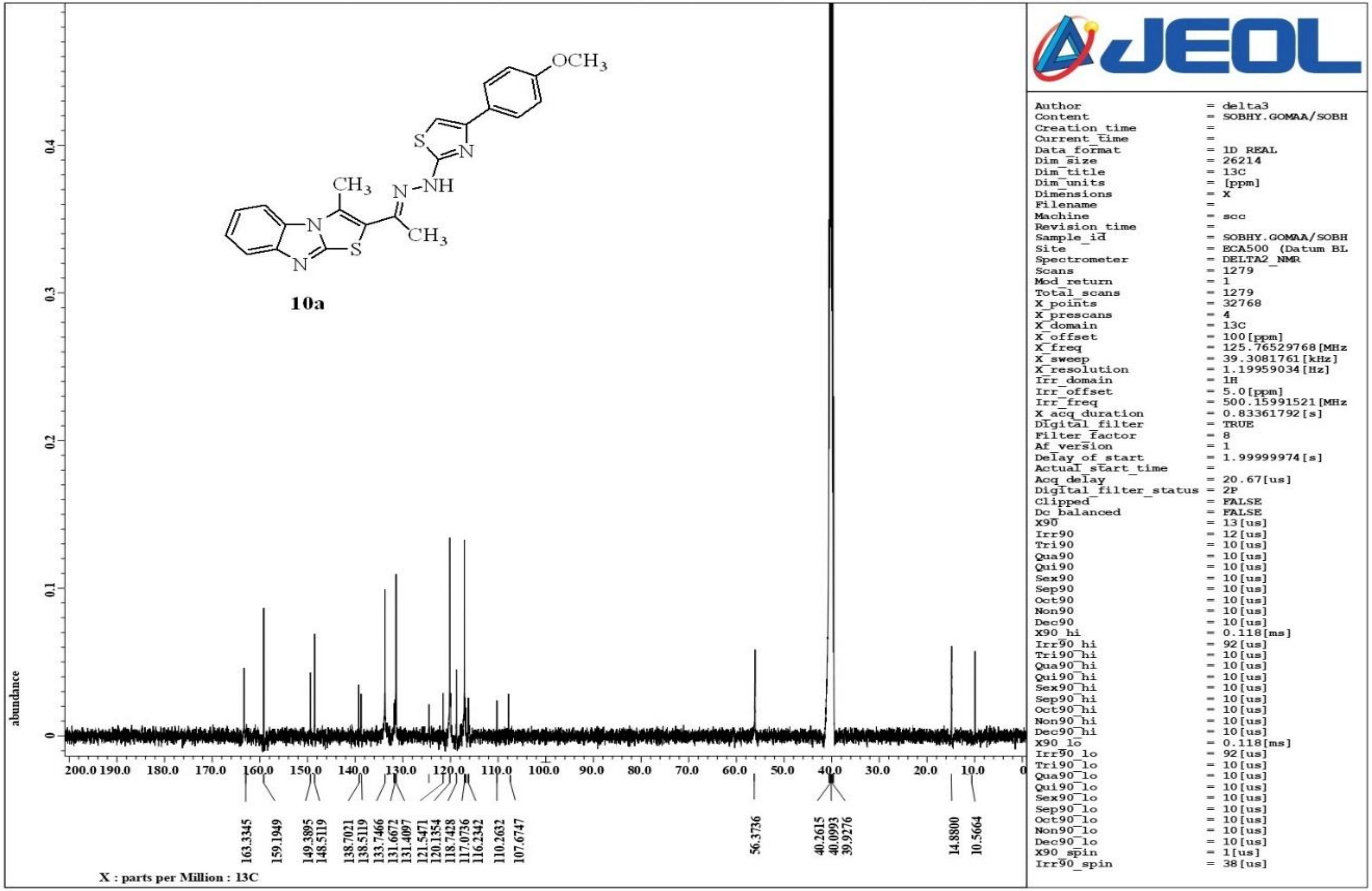

^{13}C-NMR spectra of compound **10a**

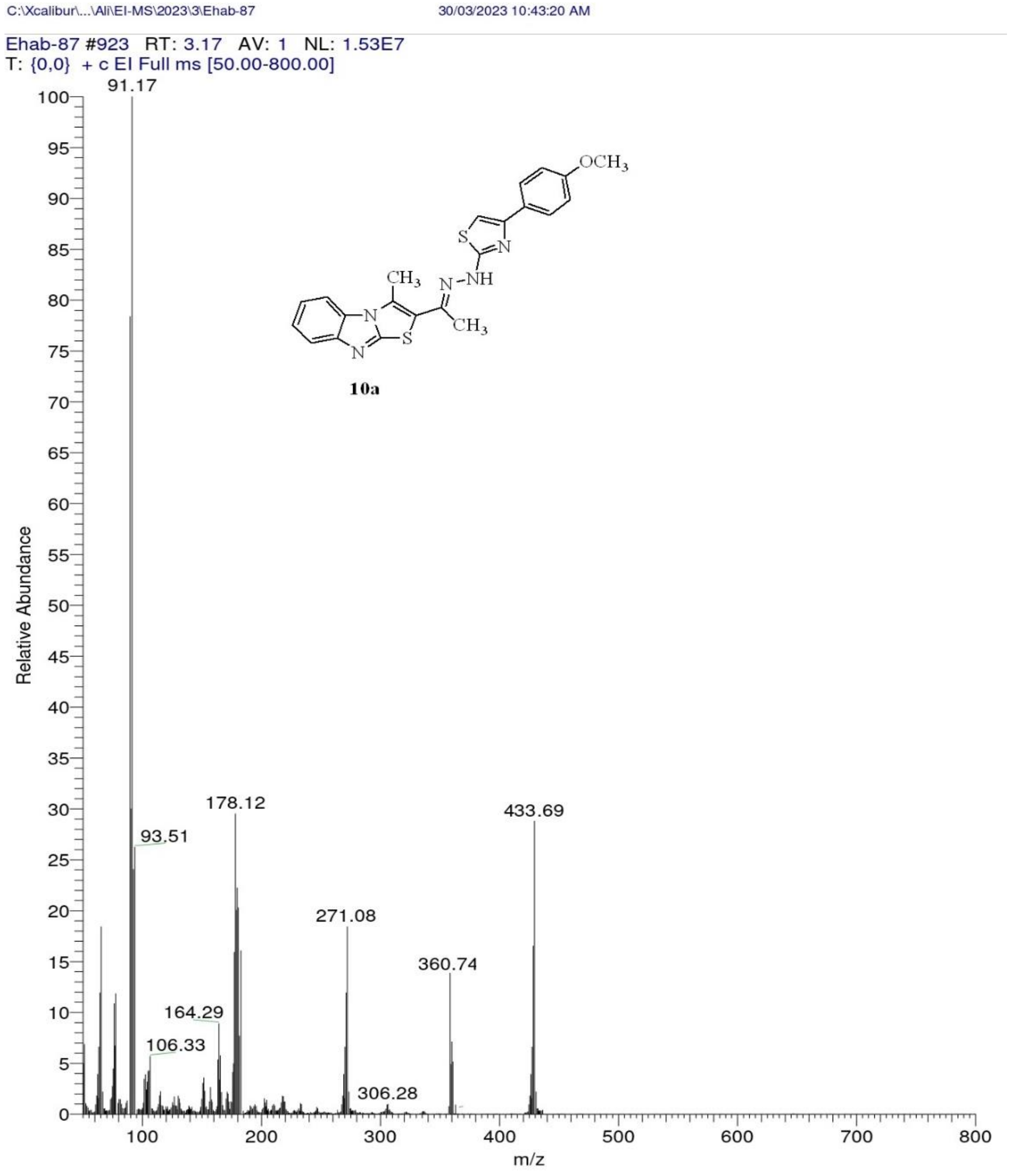

Mass spectra of compound **10a**

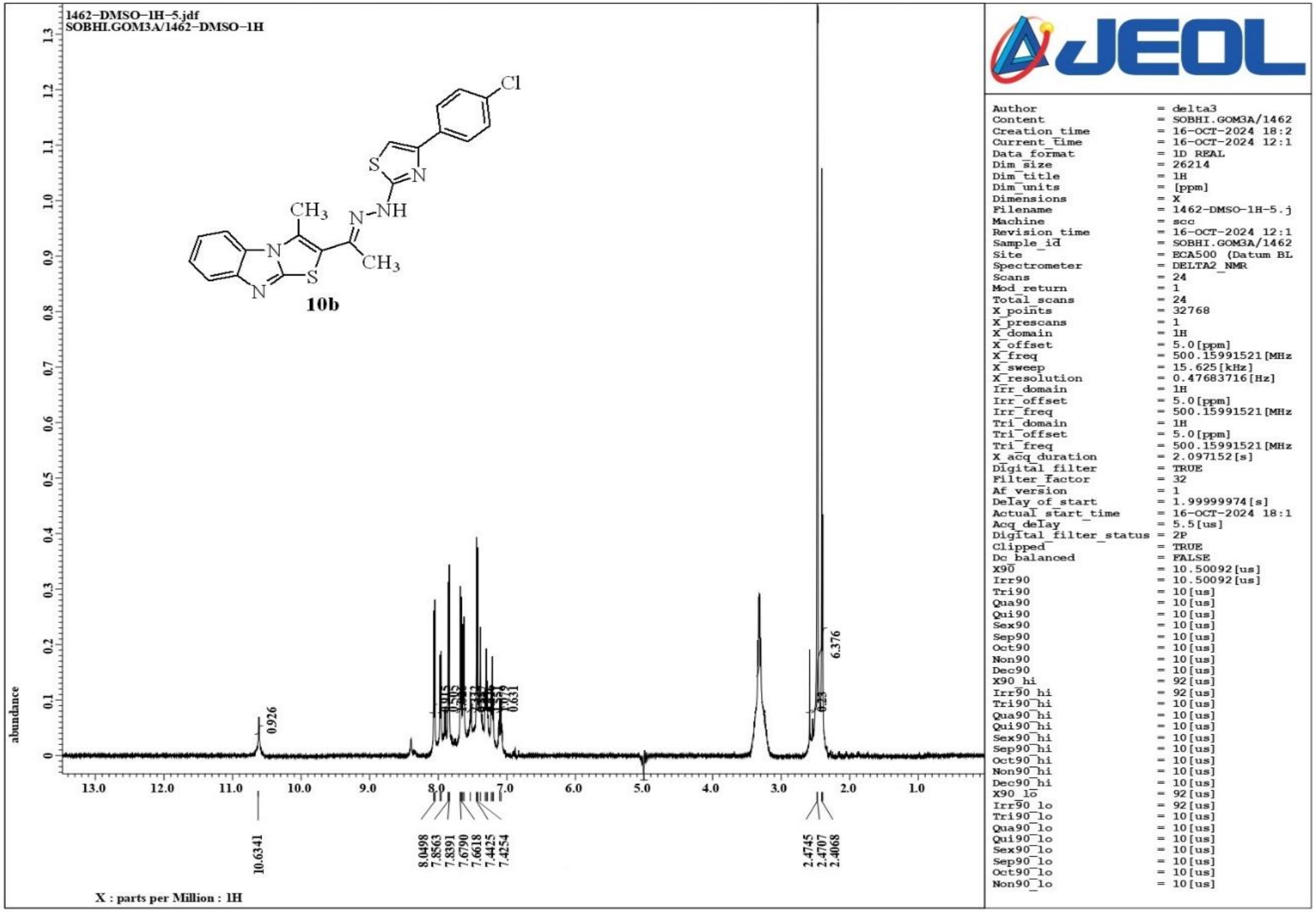

^{1}H-NMR spectra of compound **10b**

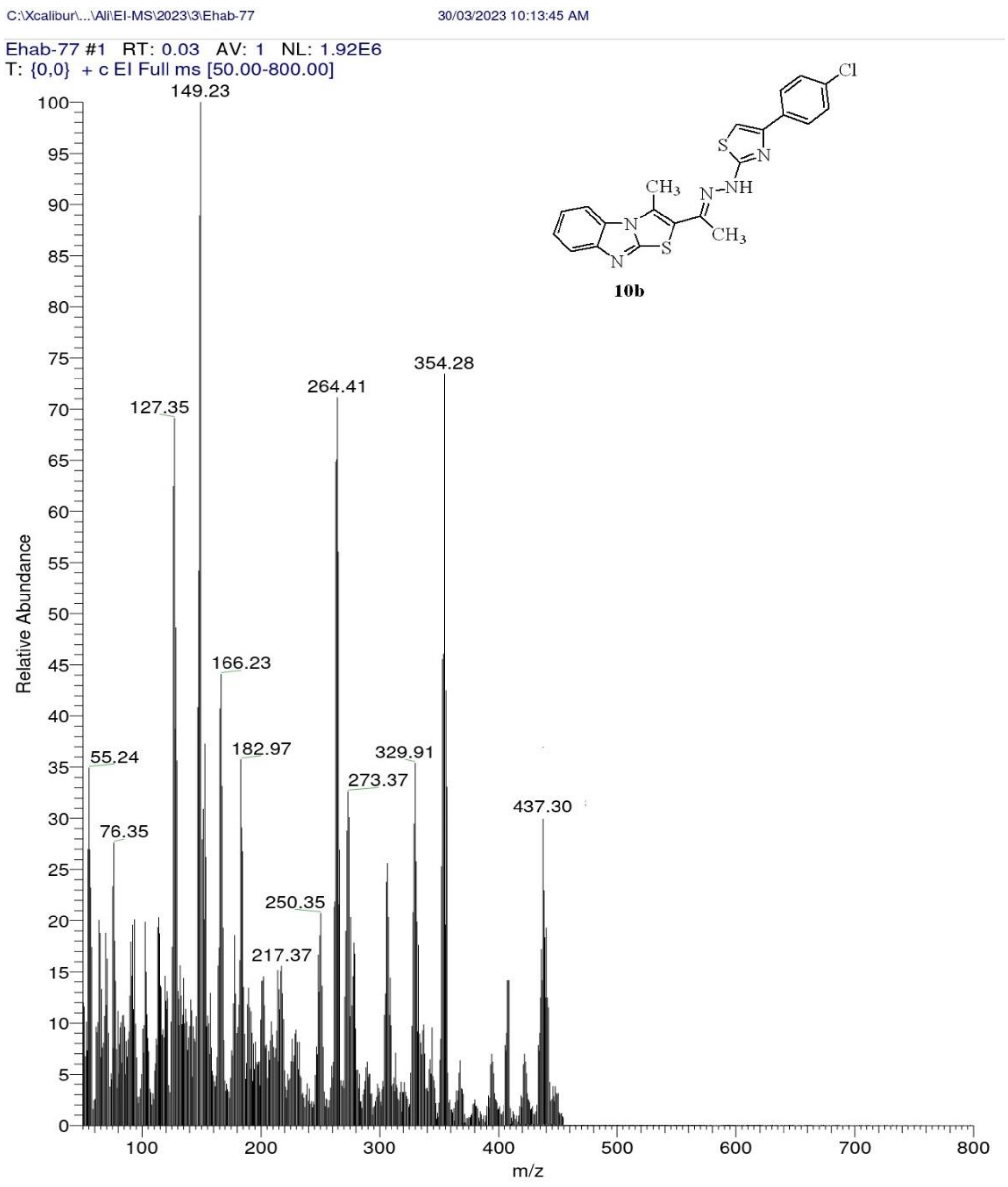

Mass spectra of compound **10b**

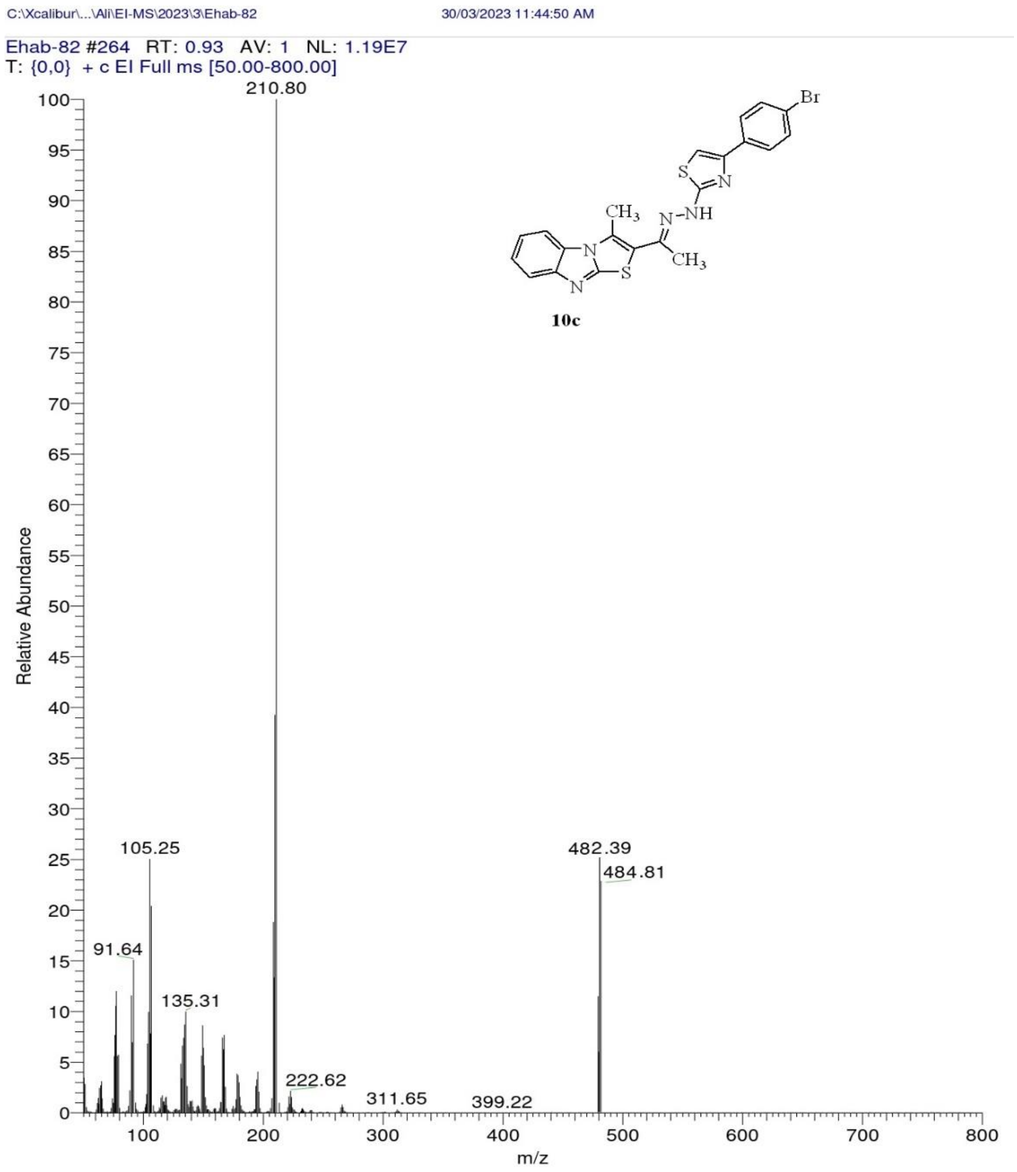

Mass spectra of compound **10c**

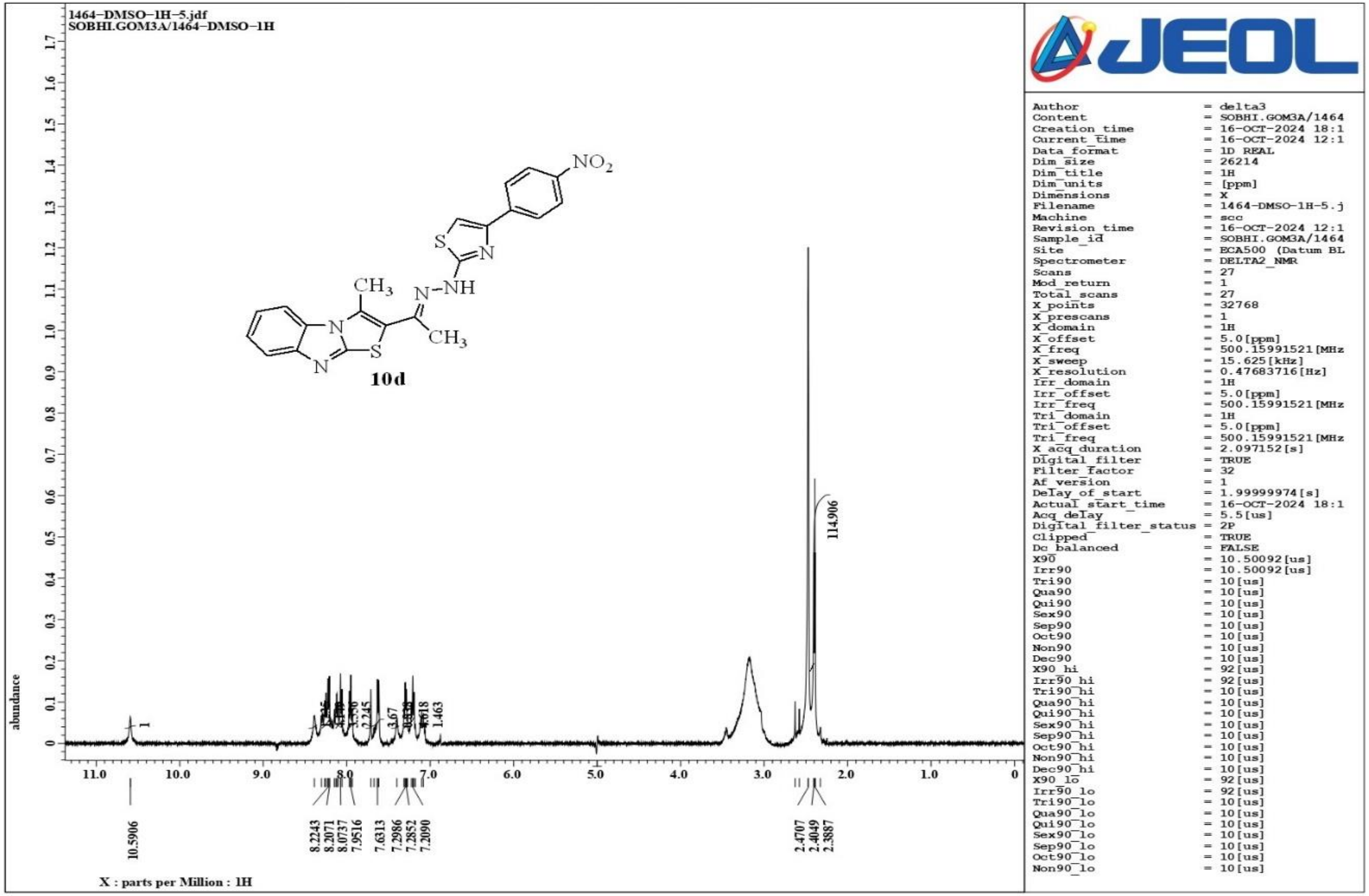

^{1}H-NMR spectra of compound **10d**

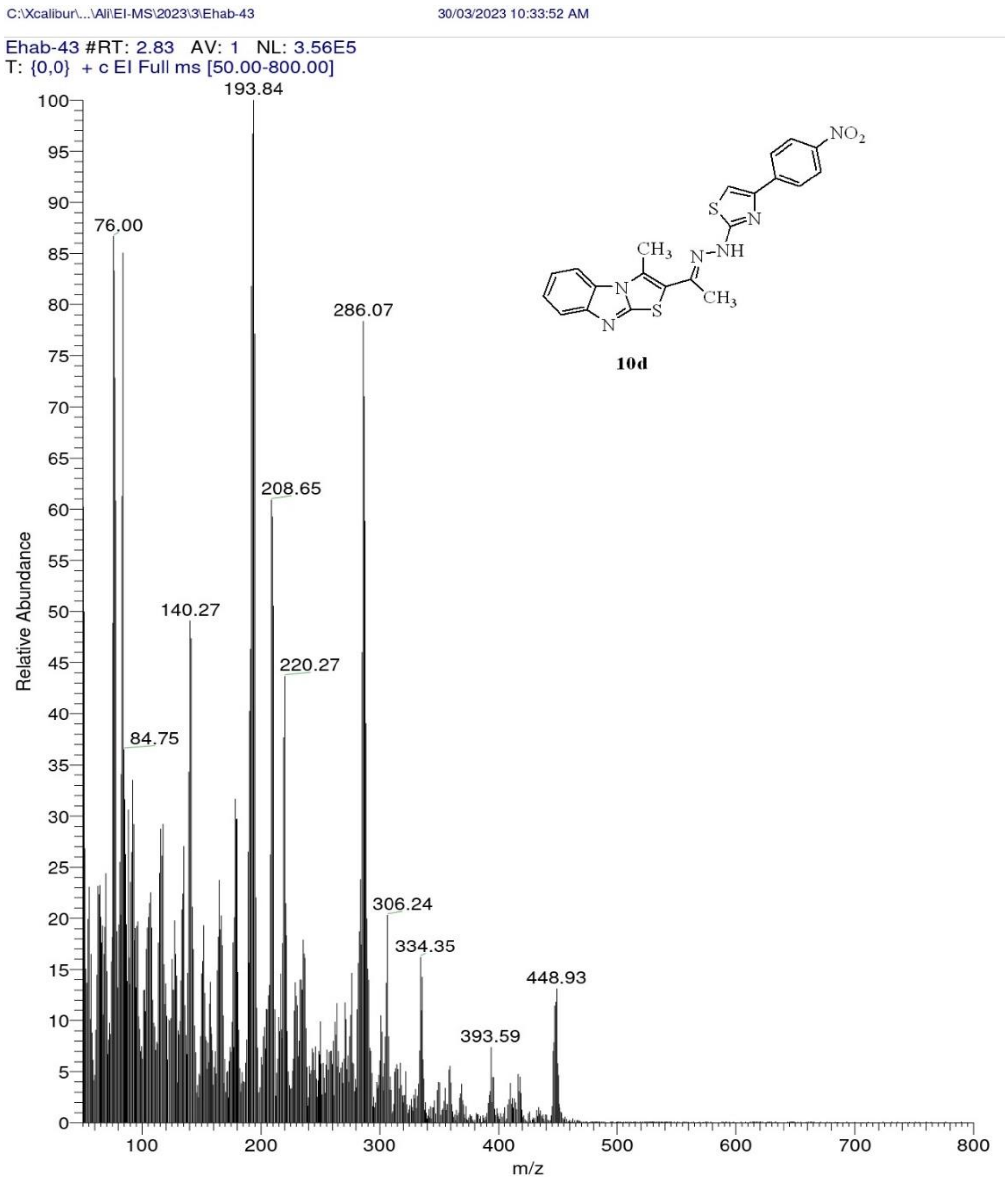

Mass spectra of compound **10d**

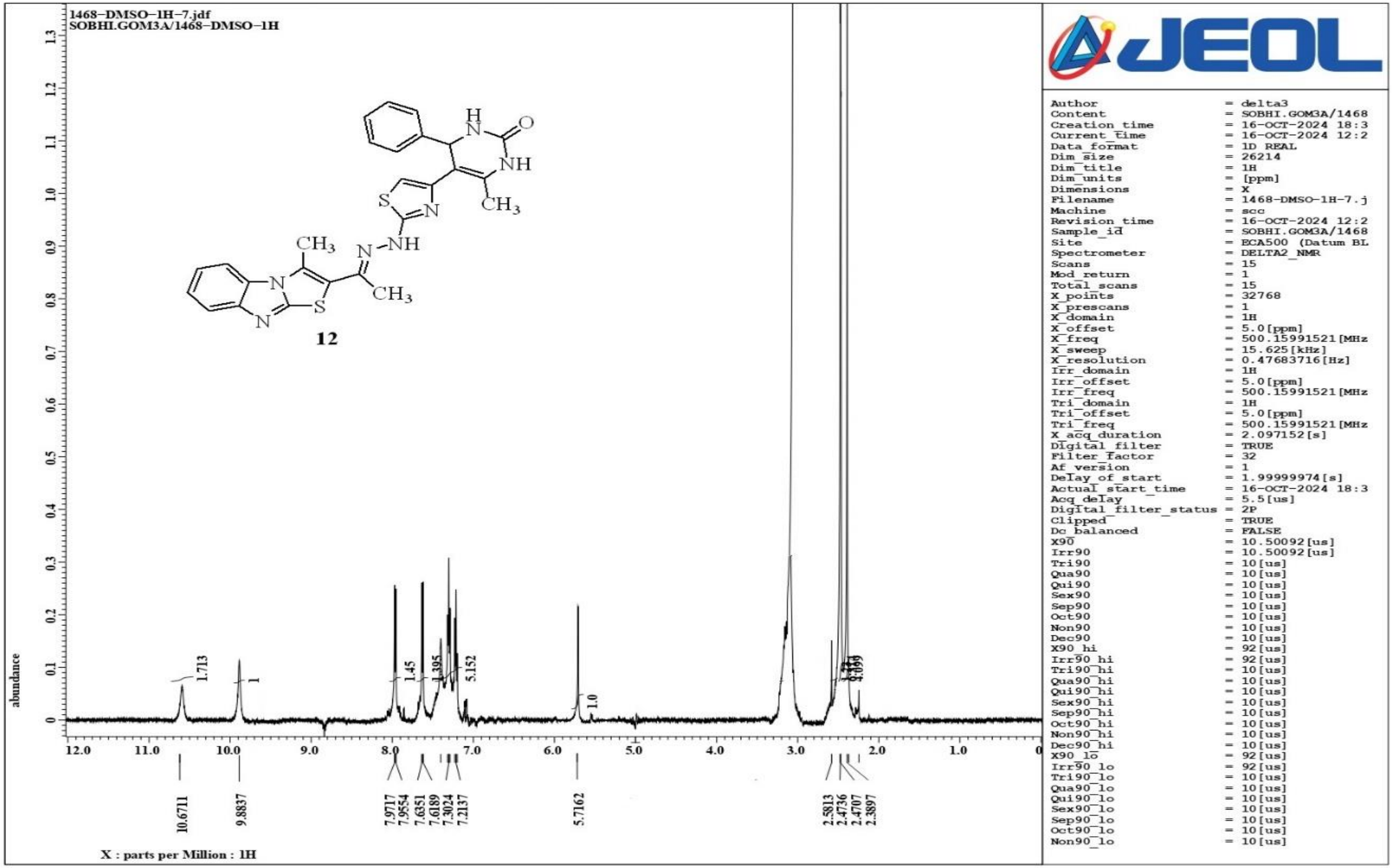

^{1}H-NMR spectra of compound **12**

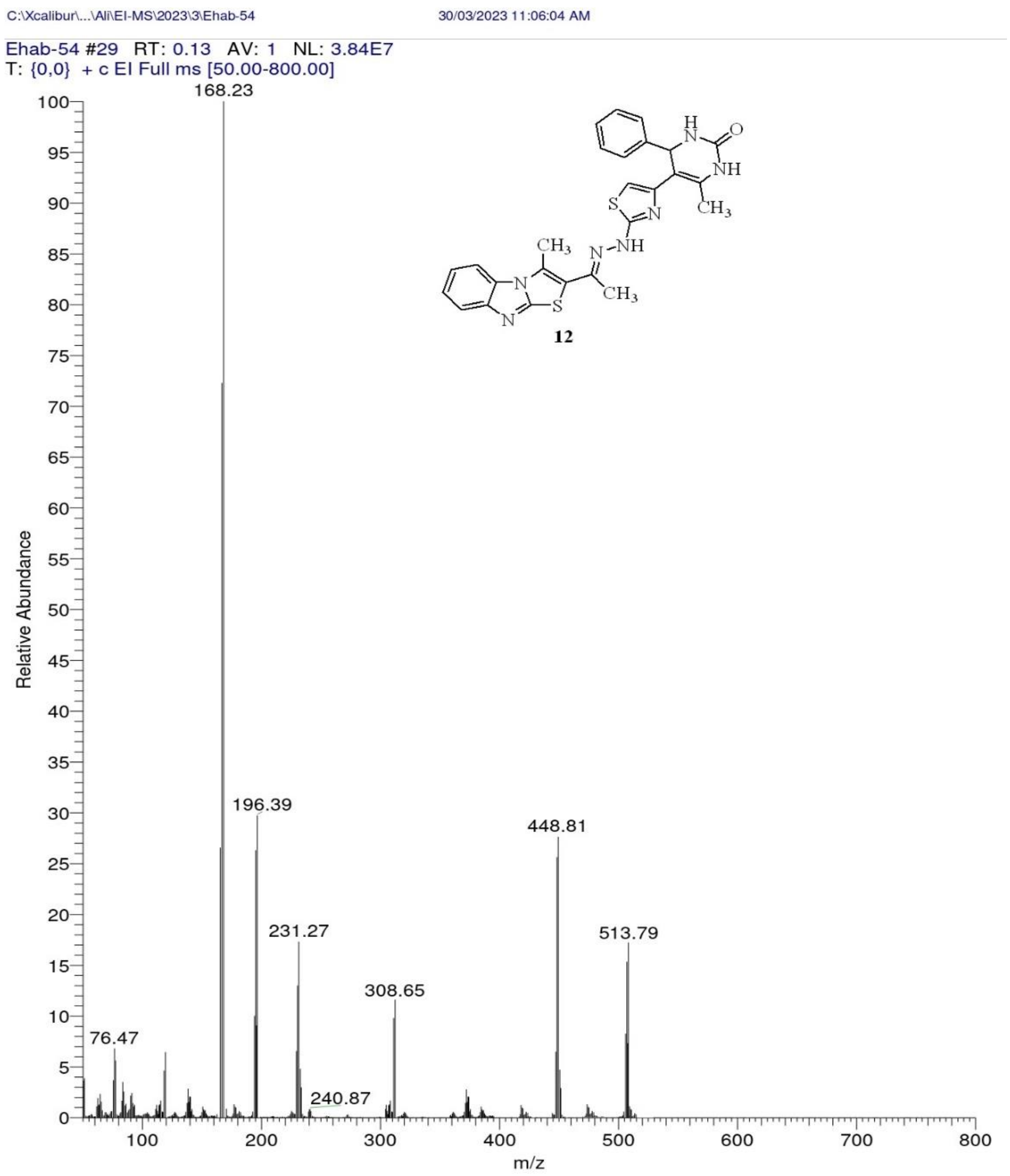

Mass spectra of compound **12**

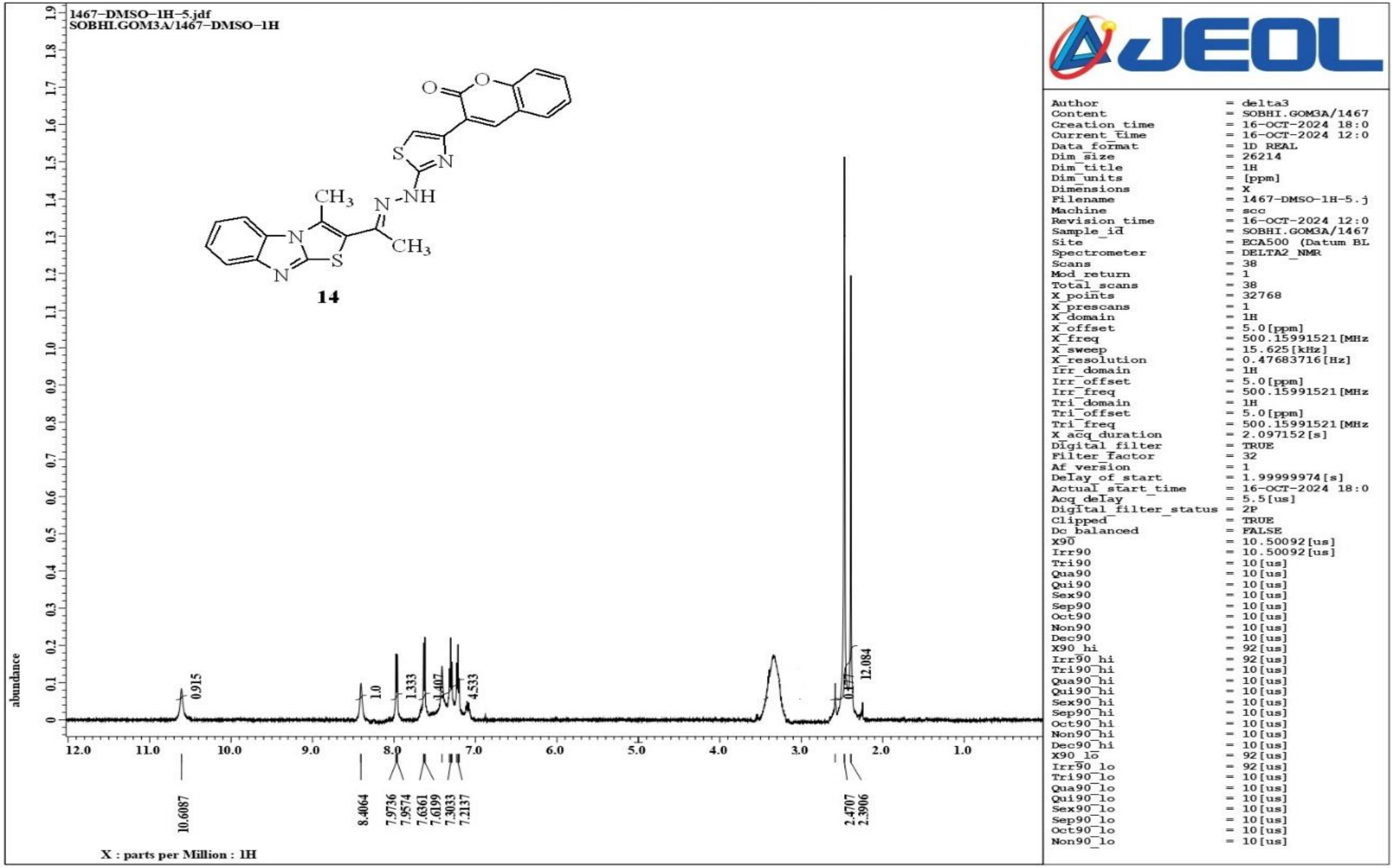

^{1}H-NMR spectra of compound **14**

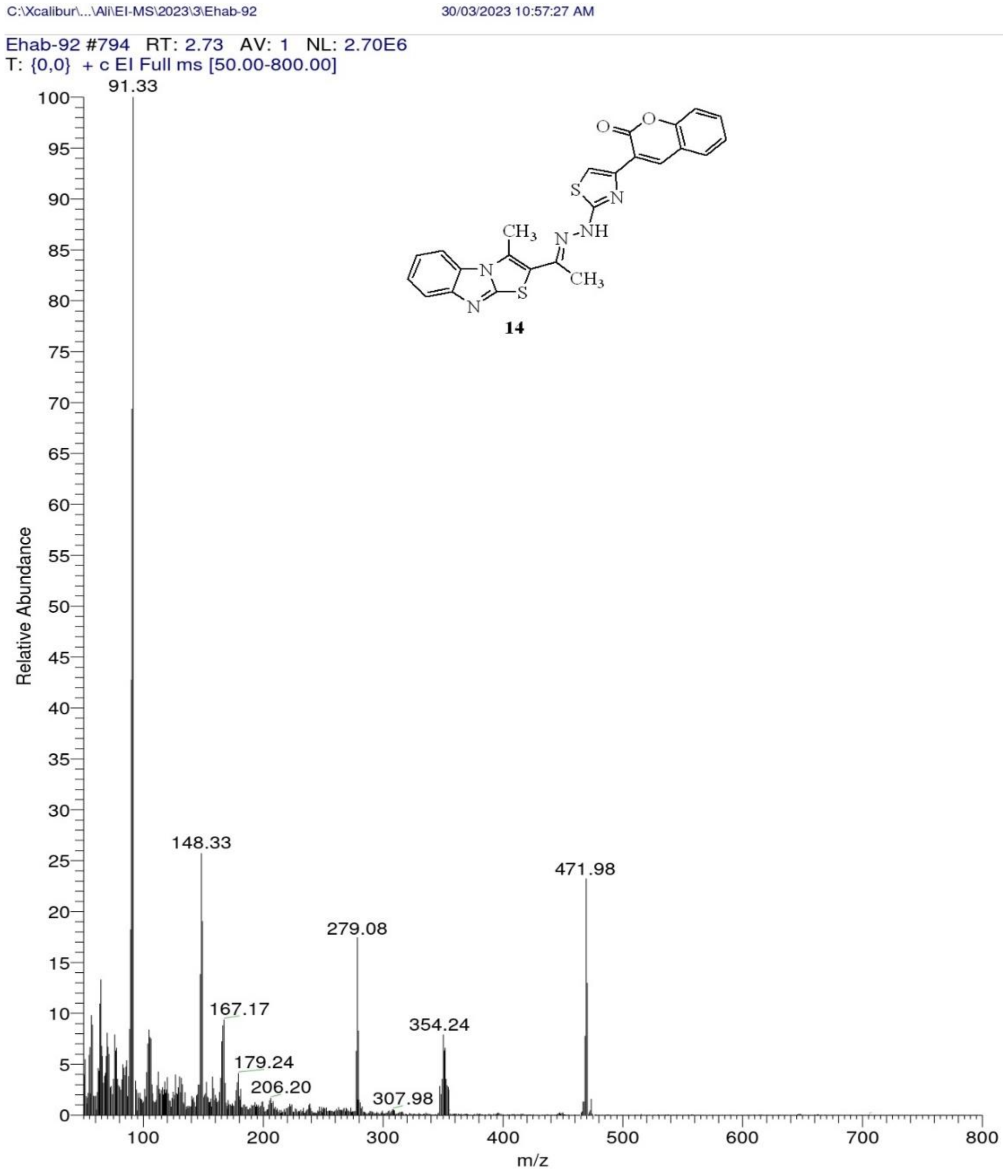

Mass spectra of compound **14**

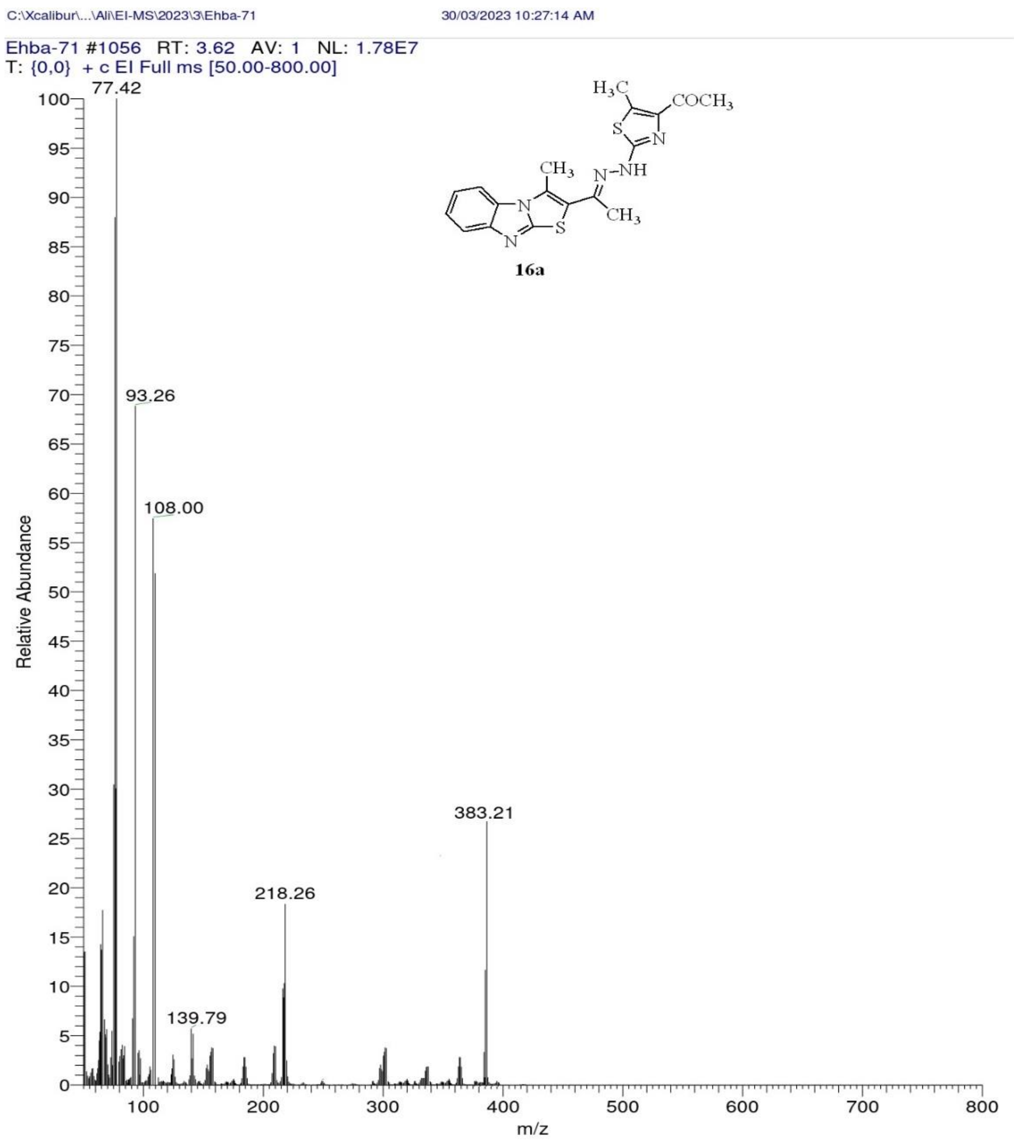

Mass spectra of compound **16a**

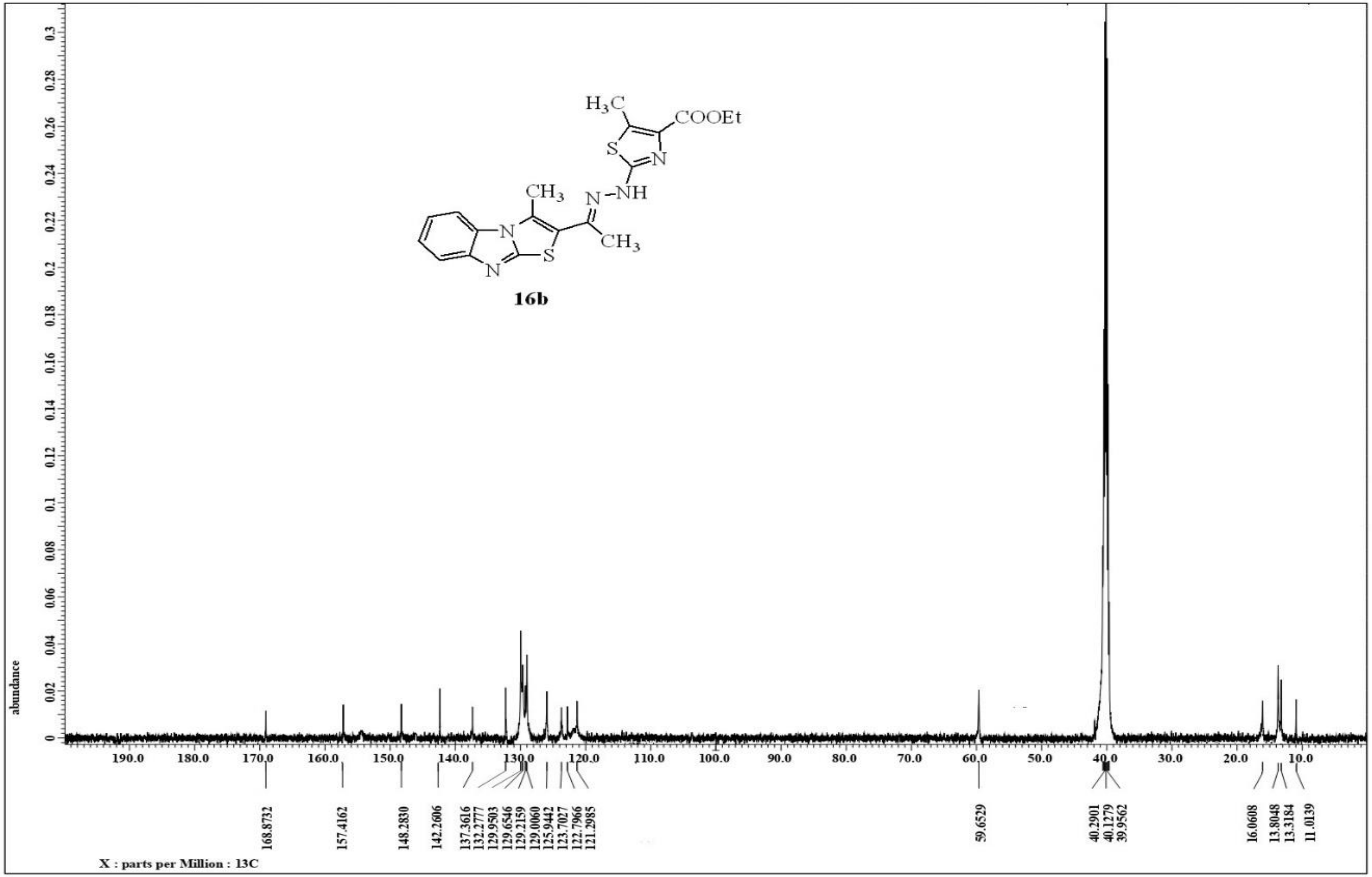

^{13}C-NMR spectra of compound **16b**

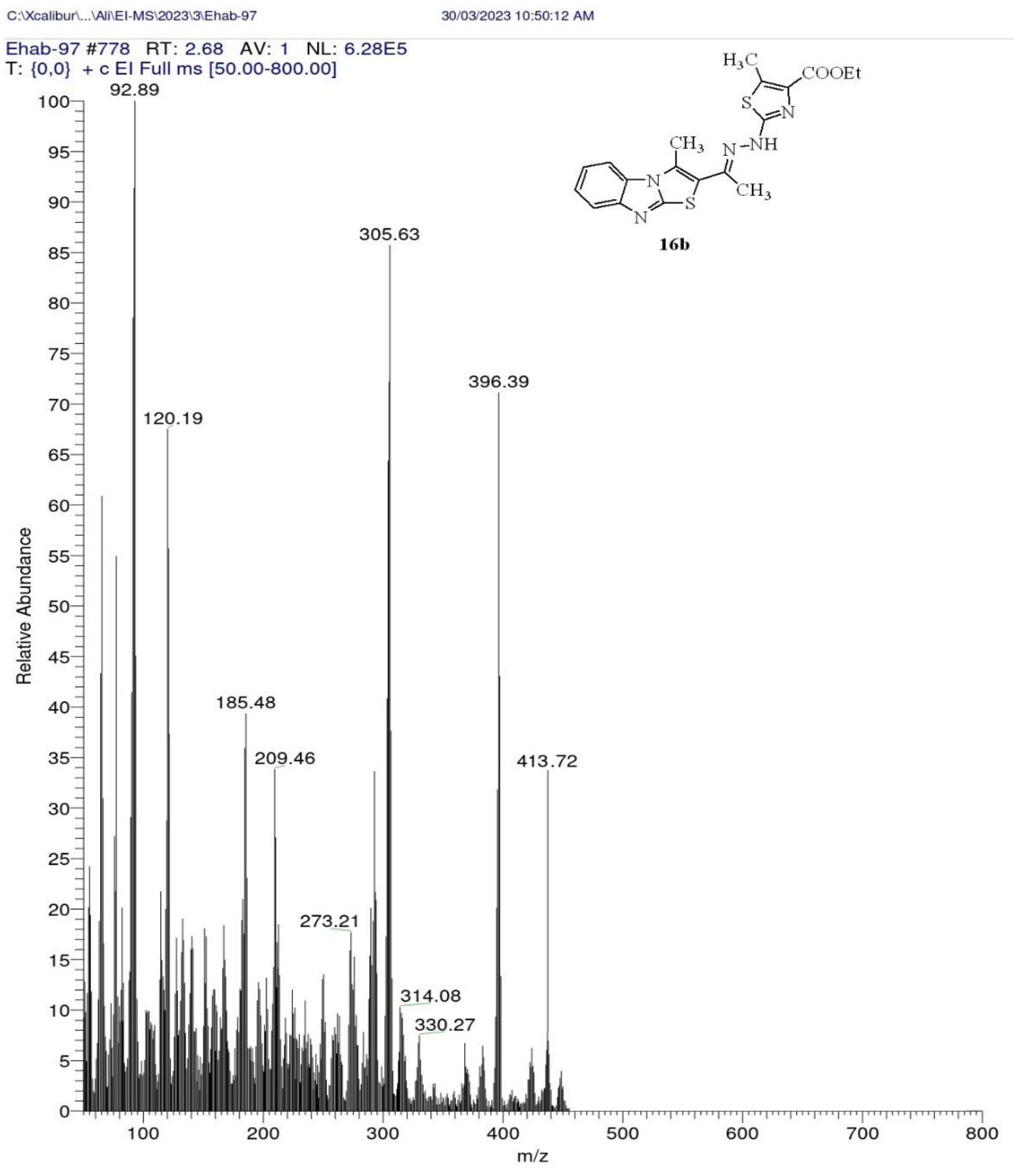

Mass spectra of compound **16b**
